# Web-based citizen science as a tool in conservation research: A case study of prey delivery by the Lesser Spotted Eagle

**DOI:** 10.1371/journal.pone.0261655

**Published:** 2022-01-26

**Authors:** Ülo Väli, Ana Magalhães

**Affiliations:** 1 Institute of Agricultural and Environmental Sciences, Estonian University of Life Sciences, Tartu, Estonia; 2 Eagle Club, Hauka, Kanepi, Põlvamaa, Estonia; Universidad Miguel Hernandez de Elche, SPAIN

## Abstract

Citizen science is increasingly contributing to ecology and conservation research, mostly by the extensive collection of field data. Although webcams attract numerous observers, they have been underused in this respect. We used prey delivery records deposited by citizen scientists in an internet forum linked to webcams to explore the diet composition and food provisioning in a forest-dwelling raptor of conservation concern, the Lesser Spotted Eagle (*Clanga pomarina*). Four pairs were studied throughout the breeding season. Most of the identified prey items were mammals (62.1%), followed by frogs (31.2%), birds (6.6%) and fish (0.1%). Among mammals, voles accounted for 84.6%, moles 12.1%, water voles 2.4% and weasels 0.4%. Frogs were the most frequently detected prey item in the spring, with a slight increase towards the end of the season, the proportion of mammals increased during the breeding season, and birds were hunted mostly in the middle of the breeding season. However, exact temporal patterns differed between nests. The food delivery rate of males increased over time but decreased somewhat before fledging the young. Females started hunting in mid-summer and their rapidly increasing effort compensated for a reduced male hunting intensity. The data collected by citizen scientists via webcams reflected the general patterns detected in earlier studies, supporting the reliability of crowd-sourced web-based data collection in avian foraging ecology.

## Introduction

The involvement of citizens from the non-scientific community in research is an increasing trend [1]. In the biological sciences, for instance, citizen scientists collect field data related to species distributions and abundance as well as macroecological trends related to global climate change [2–4]. Citizen science could contribute to conservation research [5, 6], for example, by discovering and collecting data for rare species [7]. However, data related to the breeding ecology of rare species, which is essential information for efficient conservation planning, are limited by restricted access to nest sites and other issues.

Internet cameras (webcams) are a useful tool to engage and educate public audiences in issues related to biology and conservation; hundreds of wildlife webcams are available to the general public [8]. Furthermore, special social media platforms are often linked to webcams. This enables viewers to share reactions and gain experience from discussions with fellow observers. Individuals post observations on natural history events witnessed through webcams. Such crowd-sourced data collection is potentially valuable for ecologists and, in the case of endangered species, may guide conservation actions. However, surprisingly little research has used data from webcams [9]. Examples include behavioural studies [9–11] and studies of breeding biology [12, 13]. Although webcams at nests can potentially provide detailed information on species diets, few studies have used this approach [14].

Raptors, a charismatic group of birds, have been used as model species in ecological studies and as flagship species in conservation research [15, 16]. Many raptors are threatened, emphasizing the importance of further research [16]. To educate the wider public and promote their conservation, webcams have been installed at nests of various raptor species breeding on trees, cliffs or nestboxes [9, 17].

Populations of the Lesser Spotted Eagle *Clanga pomarina*, a medium-large raptor, have been declining in several parts of its range during recent decades [18]. This forest-dwelling raptor forages in open land on various small-sized animals, such as amphibians, birds and mammals [19]. However, the relative frequencies of prey items may change during the breeding season [20] or between years [21, 22, but see 23]. Our understanding of the Lesser Spotted Eagle diet is primarily based on analyses of pellets and prey remains collected at nests [22–25]. However, this method is biased towards certain prey species (because, for example, it underestimates the proportion of amphibians [26, 27] and provides data for a short period prior to the collection of material. Direct observations and video cameras, providing unbiased information on the diet composition, are seldom used in research on the Lesser Spotted Eagle [20, 28, 29]. Yet, similarly to many other tree- and cliff-nesting raptors, webcams have been used to introduce Lesser Spotted Eagle to the wider public in several countries.

We used prey delivery records obtained from webcams to explore the diet composition of the Lesser Spotted Eagle and parental roles in food provisioning. We analysed records deposited in an internet forum by volunteers following nests of four Lesser Spotted Eagle pairs breeding in Estonia, north-eastern Europe. We first evaluated the proportions of various prey groups in the diet throughout the breeding season. According to earlier field observations [20], we expected to see a high proportion of frogs in the spring and a gradual increase in mammals in the diet over the course of the breeding season, while birds are presumably consumed at low levels. However, we also expected to see variation among studied pairs due to effects of year, location or individual choices. Second, we analysed temporal changes in parental roles by modelling trends in food deliveries at nests. In line with earlier observations [29, 30], we expected to observe the following activity patterns in all nests: 1) low number of prey deliveries by males during the incubation period with an increase in activity after the hatching of chicks, and 2) no female deliveries during incubation and small nestling periods and start of prey deliveries after nestlings reach a few weeks old.

## Materials and methods

### Study site and species

Data were collected in east-central Estonia (Jõgeva and Tartu counties), hemiboreal north-eastern Europe (S1 Fig in S1 File). Four pairs of eagles were studied, each for one successful breeding season, in various years (Table 1). Distances between nests ranged from 2.5 km to 45 km (S1 Fig in S1 File). The terrain was flat and the landscape was similar at all nest sites, including a mosaic of farmland areas of various sizes, with forests and various types of wetlands interspersed. The mean temperature during the breeding season (April–July) is 11–13°C and mean monthly rainfall is 30–90 mm (see [20, 31] for details).

**Table 1:** Main breeding events recorded at four nests of the Lesser Spotted Eagle.

Nest site	Year	Male, arrival date	Female, arrival date	Laying	Hatching	Death of nestling	Fledging
Nest 1	2012	Remo, 28 April	Tuuli, 12 April	29 April	9 June	6 August*	-
Nest 2	2013	Koit, <17 April	Eha, <17 April	3 May, 7 May	11 June, 15 June	23 June**	8 August
Nest 3	2019	Magnus, 24 April	Maia, 20 April	2 May, 7 May	11 June, 15 June	23 June**	11 August
Nest 4	2020	Indrek, 14 April	Karin, 20 April	1 May	9 June	-	5 August

*killed by a goshawk.

**older nestling killed a younger nestling.

Lesser Spotted Eagles arrive in Estonia in April and lay eggs in late April or early May; their offspring hatch in early or mid-June and fledge in early August [12]. Most eagles lay two eggs with an interval of a few days and start incubation after the first egg, resulting in asynchronous hatching. As a rule, only the older eaglet fledges, whereas the younger dies during the first weeks of life due to aggression by the sibling. Lesser Spotted Eagles use their nests repeatedly, making camera-based monitoring easy.

### Recording of events at nests

Two types of webcams (*Mobotix* and *Axis F41*) were used to record events at Lesser Spotted Eagle nests. The cameras were supplied with an external microphone and powered using solar panels, which were complemented with batteries to ensure a continuous supply of power. The webcams were installed on the nest tree (1–2 m from the nest) in early April, before the return of migrating adult birds to the breeding ground. Only in 2013, a nest was already decorated with greenery and both parents were seen on the day of webcam installation. The live broadcast was streamed via WiFi link to broadband internet continuously throughout the entire breeding season. Monitoring period was similar at all nests, ranging between 98 and 115 days (interval between the first and the last record; nest 1: 30 April to 6 August; nest 2: 17 April to 10 August; nest 3: 24 April to 11 August; nest 4: 24 April to 16 August).

Events at studied nests were recorded by voluntary observers who posted their observations in a special open internet forum (https://www.looduskalender.ee/forum/viewforum.php?f=25). The observers voluntarily joined the forum and posted observations on a voluntary basis. Forum members are aware that their observations, screen shots and videos are open source (https://www.looduskalender.ee/forum/viewtopic.php?f=7&t=17). Data collectors were not trained because data were analysed retrospectively and prey items were pooled into few well-separable classes for analysis. Moreover, the identification of prey items was validated from photos, when possible (see below). The observations covered full days, with an increasing number of recorded activities until 11 a.m. and a slow decrease thereafter (S2 Fig in S1 File). Although changes in recorded activities may be partly associated with observer activity, we believe that it mostly reflects real pattern of activities at nests, because and detected pattern is similar to the general activity pattern of spotted eagles [32] and observers originated from various countries (time zones) around the World (Kotkaklubi, unpublished data). No demographic information on forum members is available.

The parents were separated according to morphological features, mostly by specific plumage features. Sex-specific morphological characteristics were used for individual identification, as males are somewhat smaller, with thinner tarsi (appearing longer) and a narrower bill. Morphological sexing was later confirmed by behavioural observations (females laid eggs, incubated eggs most of time and cared for the young). Additionally, both parents were equipped with a GPS-transmitter and ringed with a plastic colour ring and an ornithological ring at nest 4. The male carried a transmitter and rings while the female was ringed with an ornithological ring at nest 3. This facilitated the separation of individuals in those two nests.

Spotted eagles usually carry their food in the bill, making the identification of prey items relatively easy. Prey animals were identified by observers to the species (e.g., mole *Talpa europaea* and weasel *Mustela nivalis*), genus (e.g., frogs *Rana* sp., voles *Microtus* sp. and mice *Apodemus* sp.) or class (birds and mammals) level. Seventy-two percent of records were supported with photos and thus could be validated by the authors. Approximately 85% of identifications appeared to be correct; exceptions included water voles, which were often identified as rats, and a frog identified as a salamander that is not present in Estonia. We compiled the diet composition at the class level and then separately estimated proportions of mammals, which were the most numerous groups and rather straightforward to assign to species or genera. However, prey items were frequently recorded as “unidentified small mammals”, and the share of this classification in nests was negatively correlated with the proportion of voles, the most common prey item (*r* = –0.85, n = 4), indicating that most “unidentified small mammals” were probably voles. Unidentified small mammals were pooled with voles in statistical models of differences between nests and temporal changes in prey composition. We also separated moles, which are relatively easy to identify, and, according to an earlier study [24], form an important part of the diet in Estonia.

### Data analysis

The dataset is available as supplementary information (S1 Table in S2 File). A prey delivery (i.e., the number of prey items in calculations) was used as a study unit to evaluate the diet composition and parental activity. This approach is justified by relatively low size variation among the small prey of the Lesser Spotted Eagle. Therefore, biomass was not used in statistical analyses, although it has been calculated (but has rarely been included in analyses) in earlier studies.

Statistical analyses were conducted in the statistical environment R [33]. Differences in diet composition among nests were analysed by multinomial logistic regression models (MLRs) using the R package *nnet* v. 7.3–13 [34], with nest as a dependent variable and numbers of detected deliveries of respective prey items as independent variables. Temporal changes in the relative importance of prey groups as well as temporal dynamics in prey deliveries by parents were analysed by generalized additive models (GAMs) using the R package *mgcv* v. 1.8–31 [35]. In GAMs, the number of detected deliveries of respective prey items per day was the dependent variable and Julian date was the independent variable. In the initial model, nest was used as a blocking factor (not as a random effect) because the number of nests (i.e., 4) was less than the required minimum number of levels for random factors. Moreover, monitoring effort and the number of prey items were similar among nests. Therefore, data from four nests were pooled prior to plotting the GAM smooth using the R package *ggplot2* v. 3.3.0 [36]. Additionally, GAMs for each nest were developed and compared by the visual inspection of the smooths. Given the low number of studied nests and our aim to obtain general trends, the number of knots was set to four in GAMs.

No permits were required for the study, which complied with all relevant regulations.

## Results

In total, 1451 prey items were recorded, of which 187 (12.9%; range in four nests 5.3–18.6%) were unidentified. Most of the identified 1264 prey items were mammals (62.1%), followed by frogs (31.2%), birds (6.6%) and fish (0.1%) (Table 2). Mammals outnumbered frogs in three of four nests (nests 2–4) with rather similar ratios; however, frogs were most abundant in the fourth nest (nest 1). Among mammals identified to the species level (or genera), voles formed 84.6%, moles 12.1%, water voles 2.4% and weasels 0.4% (Table 2). Voles were dominant in the diets of all pairs; however, the relative frequencies of other mammals varied remarkably. Birds had a rather low importance as prey, with substantial variation among nests. Significant differences among studied nests were detected for all main prey groups (Table 3; S2 Table in S2 File).

**Table 2:** Proportions (%, sample size in brackets) of the main prey groups (upper half) and mammal species (lower half) in the four studied nests of the Lesser Spotted Eagle. Unidentified prey items were excluded from calculations of percentages.

	Nest 1	Nest 2	Nest 3	Nest 4	Total
*All prey items*					
Fish	0.3 (1)	0 (0)	0 (0)	0 (0)	0.1 (1)
Frogs	53.7 (188)	23.7 (73)	18.8 (43)	23.9 (90)	31.2 (394)
Birds	2.3 (8)	13.3 (41)	5.2 (12)	6.1 (23)	6.6 (84)
Mammals	43.7 (153)	63.0 (194)	76.0 (174)	70.0 (264)	62.1 (785)
Unidentified item	- (80)	- (53)	- (33)	- (21)	- (187)
Total no. of prey items	430	361	262	398	1451
*Mammals*					
Mole *Talpa europaea*	14.2 (16)	32.6 (28)	20.0 (6)	2.6 (6)	12.1 (56)
Vole *Microtus* sp.	85.8 (97)	61.6 (53)	60.0 (18)	95.7 (222)	84.6 (390)
Water vole *Arvicola amphibius*	0 (0)	2.3 (2)	20.0 (6)	1.3 (3)	2.4 (11)
Hare *Lepus* sp.	0 (0)	1.2 (1)	0 (0)	0.4 (1)	0.4 (2)
Weasel *Mustela nivalis*	0 (0)	2.3 (2)	0 (0)	0 (0)	0.4 (2)
Unidentified small mammal	- (40)	- (108)	- (144)	- (32)	- (324)
Total no. of mammals	153	194	174	264	785

**Table 3:** Estimates (±SE) of multinomial models indicating significant differences among three studied pairs (compared with nest 3).

	Nest 1	Nest 2	Nest 4
(Intercept)	‒0.24 ± 0.14	‒0.08 ± 0.13	‒0.30 ± 0.13 *
Frogs	0.77 ± 0.14 ***	0.25 ± 0.16	**0.41** ± 0.15 **
Birds	‒0.42 ± 0.45	0.89 ± 0.32 **	0.38 ± 0.34
Voles	‒0.14 ± 0.08	‒0.13 ± 0.08	0.15 ± 0.07 *
Moles	0.82 ± 0.46	1.09 ± 0.43 *	‒0.35 ± 0.55
Other	‒1.72 ± 1.08	‒0.36 ± 0.59	‒0.43 ± 0.63

Significant differences are indicated in bold

*P < 0.05

**P < 0.01

***P < 0.001.

**Fig 1:**
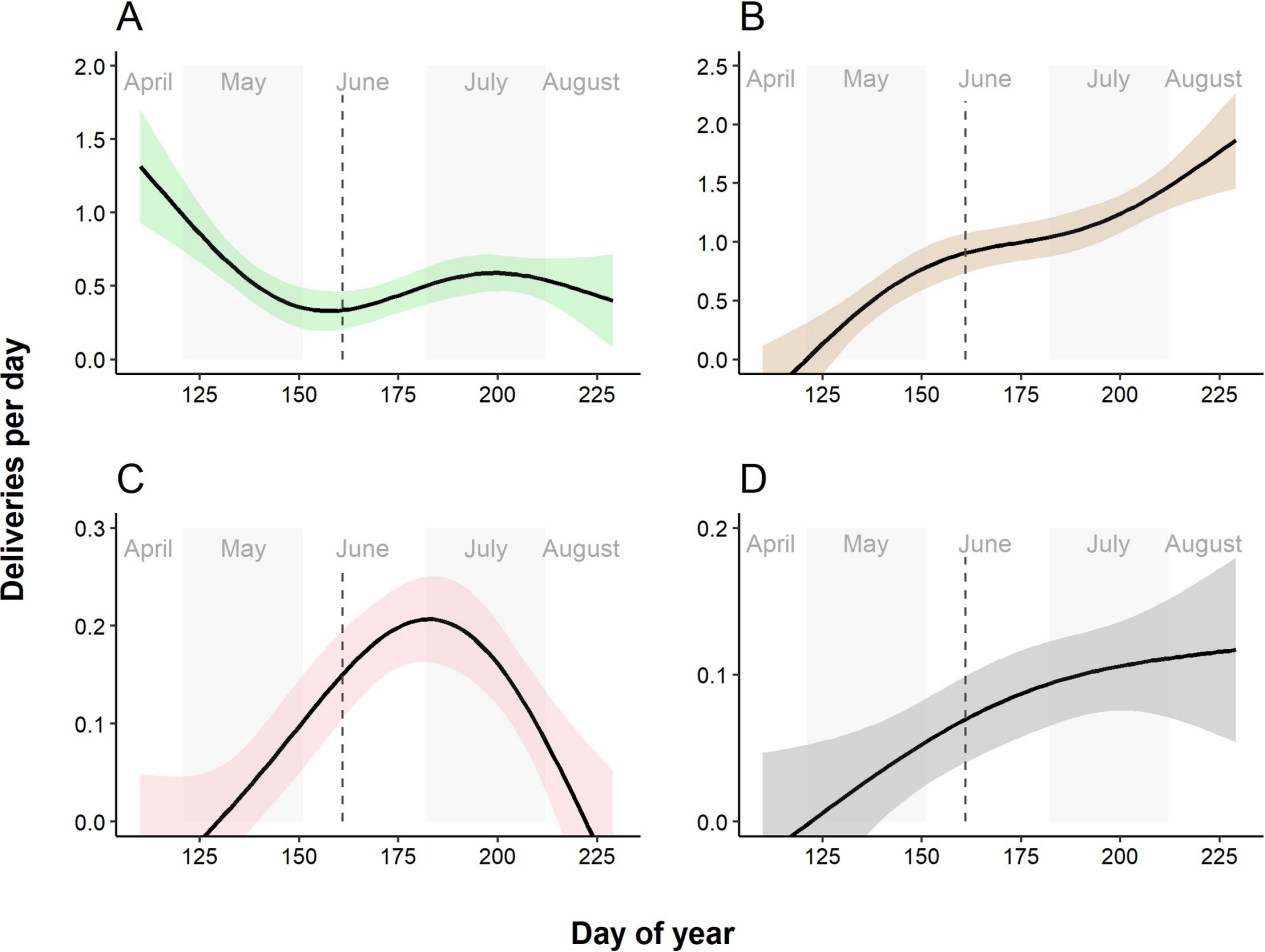
General additive models indicating the relative importance of frogs (A), voles (B), birds (C) and moles (D) in the diet of the Lesser Spotted Eagle. 95% confidence intervals are shaded. Hatching time is indicated by a dashed vertical line. Note the differences in scales on the Y-axis.

Date significantly influenced the number of prey items delivered per day. Frogs had the highest importance in the spring and increased somewhat in the diet towards the end of the season (Fig 1). The proportion of mammals increased over the course of the breeding season. Birds were hunted mostly in the middle part of the breeding season. These general trends were noticed in each studied pair separately, although the exact patterns differed among nests (S3 Fig in S1 File).

**Fig 2:**
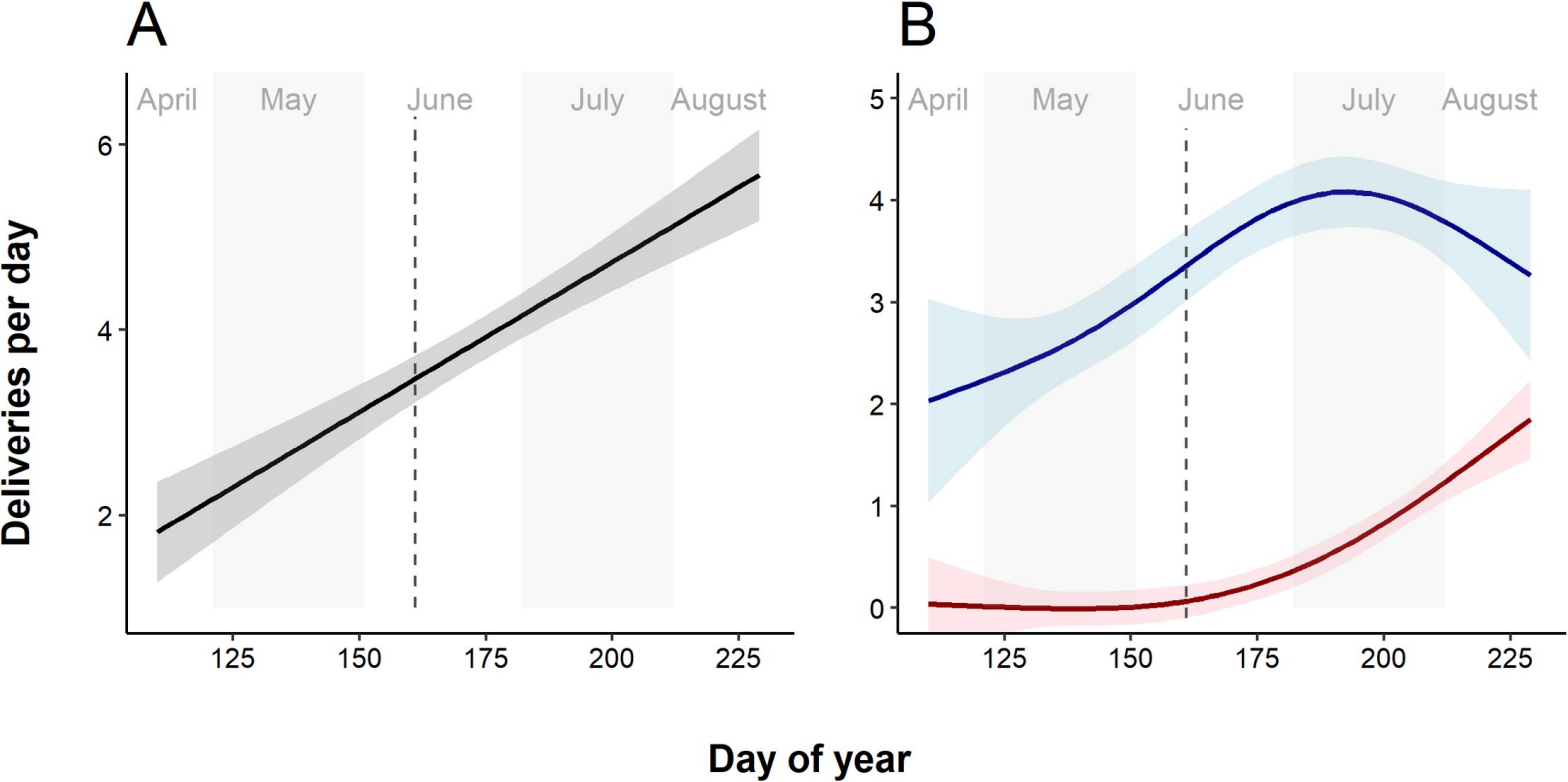
General additive models indicating temporal changes in prey deliveries in total (A) and in male (blue) and female (red) parents (B). 95% confidence intervals are shaded. Hatching time is indicated by a dashed vertical line.

The total number of prey items increased over time (linear trend: *β* = 0.03, *R*^*2*^ = 0.15, *F*_*1*,*375*_ = 68.5, *P* < 0.001; Fig 2). The food delivery rate of males increased over time with a slight decrease before fledging the young. Females started prey deliveries in the middle of summer and their rapidly increasing effort compensated for a lower number of deliveries by males. However, this general pattern involved very different temporal dynamics of prey deliveries by the two parents in individual nests (S4 Fig in S1 File). Yet, even in July and August, when both parents hunted, males brought 80% of prey (n = 751, all nests pooled). During these months, males delivered marginally more mammals (63%) than females (57%; χ^2^_1_ = 3.4, *P* = 0.06), while females brought more frogs (35%) than males (24%; χ^2^_1_ = 6.3, *P* = 0.01).

## Discussion

Citizen scientists recorded valuable data on diet composition and dynamics, changes in food delivery by parents, and breeding events. Most of the identified prey items were mammals (mainly voles) and frogs, while other prey groups were hunted occasionally. However, the relative importance of each prey group changed over the course of the breeding season. Similarly, food delivery rates by the male and female parents changed gradually. However, exact temporal patterns differed between nests.

### Diet composition and changes during the breeding season

Voles are a staple food across most of the Lesser Spotted Eagle range [24, 25, 37–40]. The results of these pellet-based studies were confirmed by our analysis based on webcams. Yet, there are regional differences in diet. For example, a pellet-based study has shown that moles are an important component of the diet in Estonia [24]. Our camera-based study indicated a much lower proportion of moles in the same region, but the proportion differed remarkably among nests. Similarly, water voles seemed to compensate for *Microtus* voles in some nests.

The proportion of amphibians was higher than recorded in earlier pellet-based studies. Frogs formed one-third of prey items, on average, but accounted for more than half of the diet in one of the study nests. In Estonia, frogs depart their wintering sites in mid-April and spawn in shallow ponds and ditches in late April. At that time, they are most visible and available to predator; later, they spend more of the daytime hiding. Consistent with an analysis of field records in foraging grounds [20], based on webcam data, we observed the replacement of frogs with mammals and birds over time, with two nests showing an increase in the proportion of frogs late in the season. Likewise, Palášthy & Meyburg [28] observed a decrease in frogs in the diet as nestling period proceeded in central Europe. The abundance of small mammals typically increases steadily from the spring to late summer or autumn [41], consistent with the diet of the Lesser Spotted Eagle. The proportion of voles is highest in August when Lesser Spotted Eagles forage on harvested fields [30]. The proportion of birds in the diet peaked in mid-summer when they are most abundant after offspring fledging. Indeed, bird fledglings are an easy target and therefore a staple food in mid-summer for many generalist raptors [41]. In summary, eagles obtained increasingly energy-rich prey during the season; although this pattern is expected based on the increasing energy requirements of nestlings, we propose that it mostly reflects changes in the temporal availability of particular prey types [42–44].

Obviously, we were unable to detect all of the prey consumed by the species. Often, parents bring larger prey to the nest and consume smaller items themselves at capture sites [45]. Hence, our study was restricted to diet composition of nestlings and females during incubation and the early nestling phase. Interestingly, the four studied pairs differed remarkably with respect to diet composition and temporal dynamics. Unfortunately, our analysis based on a small number of nests across different years did not provide sufficient resolution to distinguish year-effects from individual and regional differences. Future monitoring of the same nests over several years, supplemented with data on weather conditions and prey abundance, would enable the exploration of links between environmental factors and diet composition. Additionally, using biomass instead of number of prey items would provide deeper insight into energetic constraints connected with temporal changes in diet composition.

### Parental roles in the Lesser Spotted Eagle

In birds, mates often adopt different parental roles [46]. In most raptor species, the female incubates the eggs and looks after the young and the male provides food, with assistance by the female after the young are half-grown [41]. The Lesser Spotted Eagle follows this pattern [29], consistent with the result of the current study, thereby supporting the reliability of data (and individual identification) collected by citizen scientists via webcams.

Our study was limited to events at nests. Male raptors often deliver prey to females outside of the nest [41]; in these cases, the female delivers prey items to the nest but the prey is hunted by the male. Although detailed studies are lacking, this has been reported in Lesser Spotted Eagle pairs [29], particularly during the early nestling stage. This, however, does not affect the relative frequencies of parental provisioning because males often brought prey directly to the nest (Fig 2B). Moreover, in the closely related Greater Spotted Eagle *Clanga clanga*, hunting by females in the vicinity of nest has been observed [31]. The importance of female hunting is further supported by telemetry studies and direct field observations (Ü. Väli, unpubl. data).

Increased food consumption during the nestling period, peaking in the late nestling stage, has been observed in several raptor species; remarkable differences among individual nests have been recorded in these species [41]. The Lesser Spotted Eagle followed this general pattern. An increase in delivery rate is essential to satisfy the growing energy demands of developing offspring. However, the rate of food provisioning may decrease at the end of the nestling period to support fledging by decreasing weight and increasing exploratory activity. This has been noticed, for example, in the Golden Eagle *Aquila chrysaetos* [47]. We detected a late decrease in the delivery rate in only one of four studied nests, with an increasing trend otherwise. However, in two of four nests, males showed a decreased delivery rate, resulting in a slight decrease in the general trend. Interestingly, no significant differences in delivery rate were detected in the sister species Greater Spotted Eagle [31].

### Webcams as a non-invasive method and citizen science

Citizen science provides an opportunity to conduct long-term research at broad geographic scales, which are impossible to sample extensively by traditional field research models [2]. Webcams, in turn, have been used to evaluate small numbers of sites in detail, with the time expenditure shared by many people. A large number of observers enables the intensive, nearly continuous sampling of events. This may result in rather large sample sizes, enabling detailed analyses of events and processes. However, one should keep in mind that the number of independent sample sets is low (for example, nest-cams follow single nests). Collaboration and the compilation of similar independently collected data sets for the same subject would help to overcome this obstacle and increase the generalizability of results. Yet, despite the low number of monitored nests, our results clearly demonstrate the advantages of camera-based surveys that enable assessments of bird behaviour without observer interference and provide unbiased recording of prey items brought to nests.

One of the main concerns in citizen science is the variable experience of observers, potentially affecting data quality [1, 2]. The data collected by citizen scientists via webcams reflected the general pattern detected in earlier studies, supporting the reliability of crowd-sourced data collection (see also [9]). Although the experience of citizen scientists in species identification varied, experienced observers often guided less experienced forum members. Still, the ratio of voles and unidentified small mammals indicated variation in identification precision among nests. In addition to changing observers and experience levels, the location of the camera determines the precision of identification. Finally, nearly three-quarters of recorded prey items were validated by supporting photos and most were correctly identified. In conclusion, webcam-based citizen science is a reliable source for information on the diet of raptors. Given the increasing use and popularity of webcams, such crowd-sourced data collection has high potential for ecological research in the future.

## Supporting information

S1 File Supplementary Figs 1–4. Location of studied nests; temporal distributions of recorded activities; temporal changes in relative importance of prey groups and in prey deliveries at four studied nests.
(PDF)

S2 File Supplementary Tables 1, 2. Data used in the analysis; multinomial models indicating dietary differences among studied pairs.
(PDF)
