# Supplementary material for: Web-based citizen science as a tool in conservation research: A case study of prey delivery by the Lesser Spotted Eagle
Source: PLoS One. 2022 Jan 26;17(1):e0261655. doi: 10.1371/journal.pone.0261655 (PMC8791511; doi:10.1371/journal.pone.0261655)
Supplement: S1 File — Location of studied nests; temporal distributions of recorded activities; temporal changes in relative importance of prey groups and in prey deliveries at four studied nests. (PDF) [file pone.0261655.s001.pdf]

Web-based citizen science as a tool in conservation research: a case study of prey delivery by the Lesser Spotted Eagle

Ülo Väli*, Ana Magalhães

Supporting Information S1 Figures

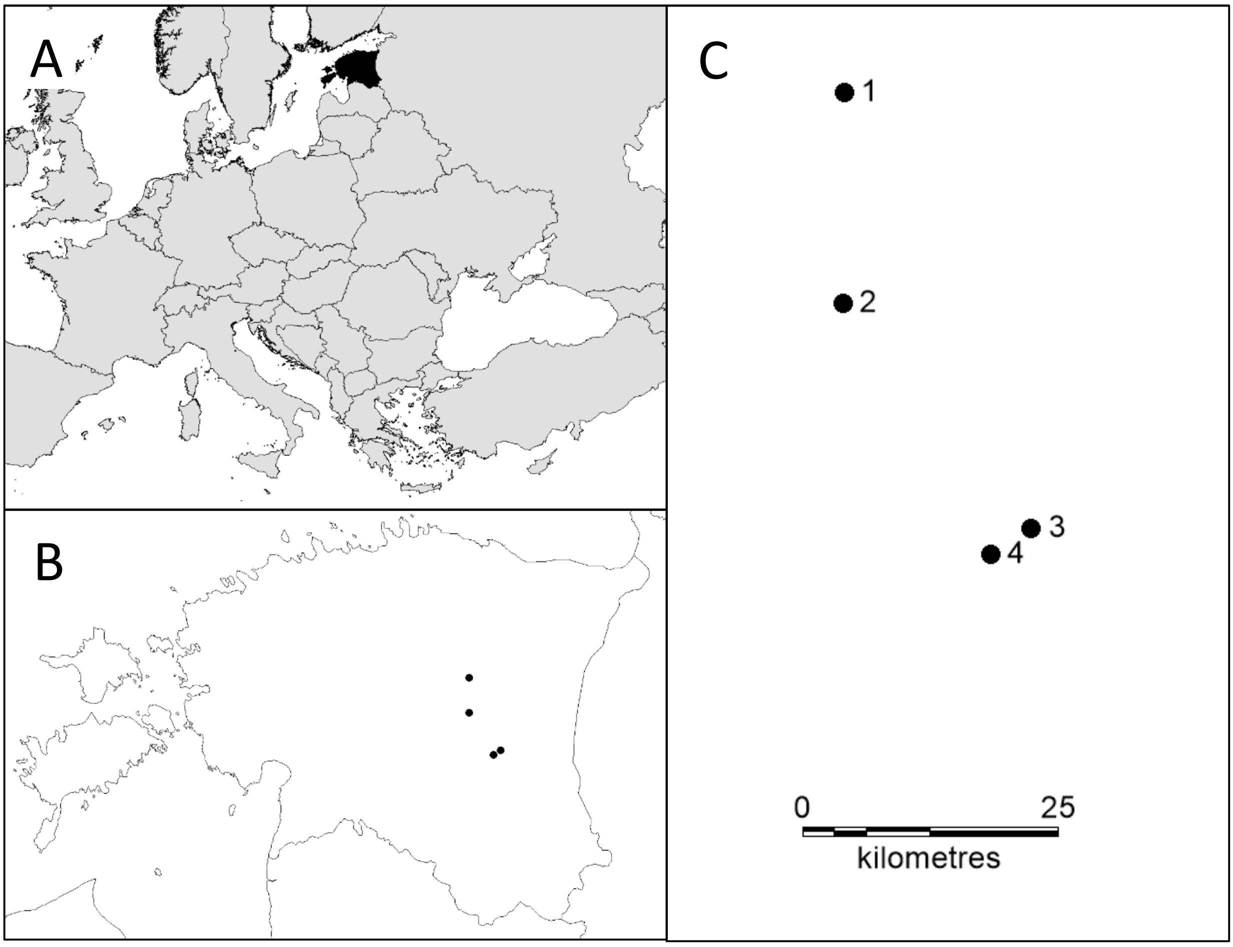

Supplementary Figure 1. Location of studied nests in Europe (A), Estonia (B) and in relation to each other (C).

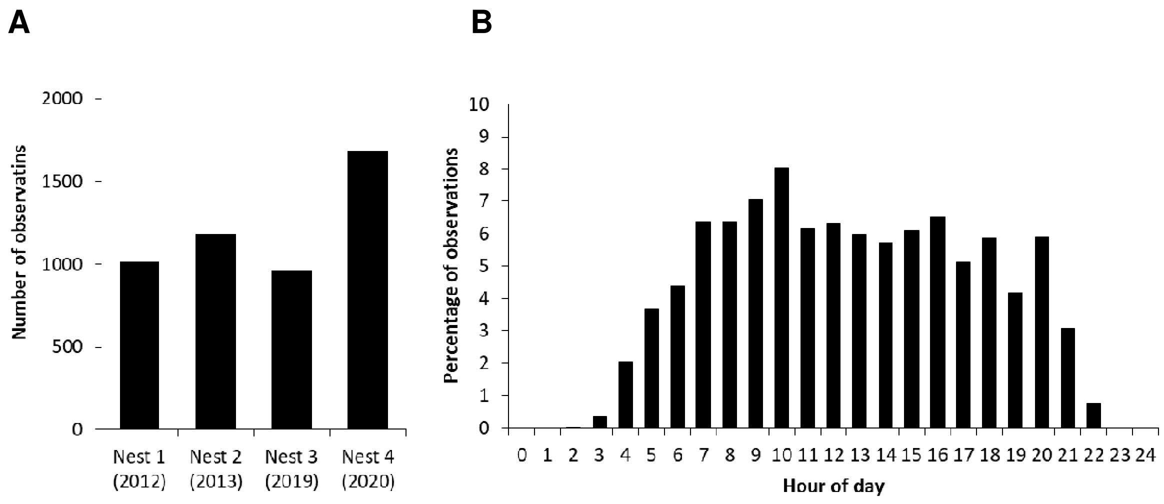

Supplementary Figure 2. Number of recorded activities (all activity types; **A**) and daily distribution of activities (average of four study years, local time UTC+3; **B**) at nests recorded by voluntary observers.

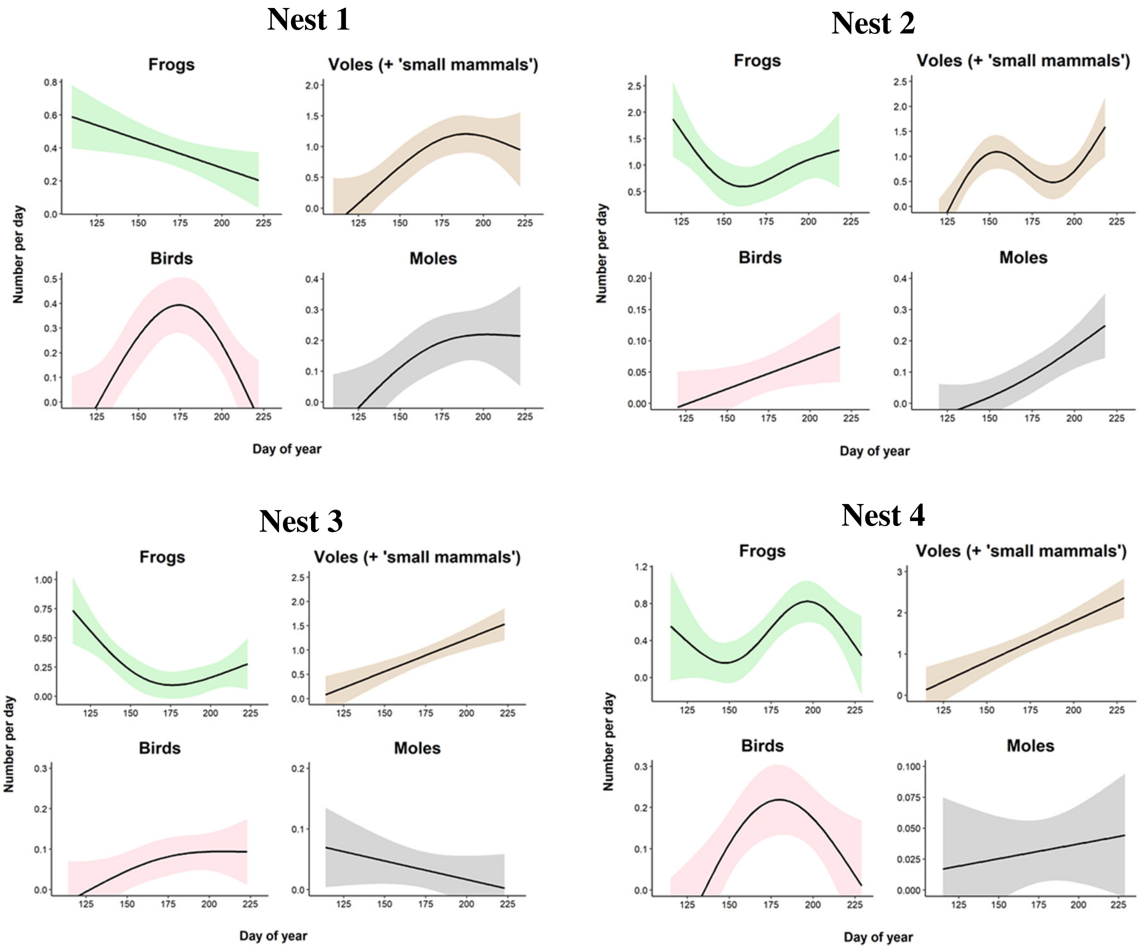

Supplementary Figure 3. General additive models indicating the relative importance of prey groups in four studied nests.

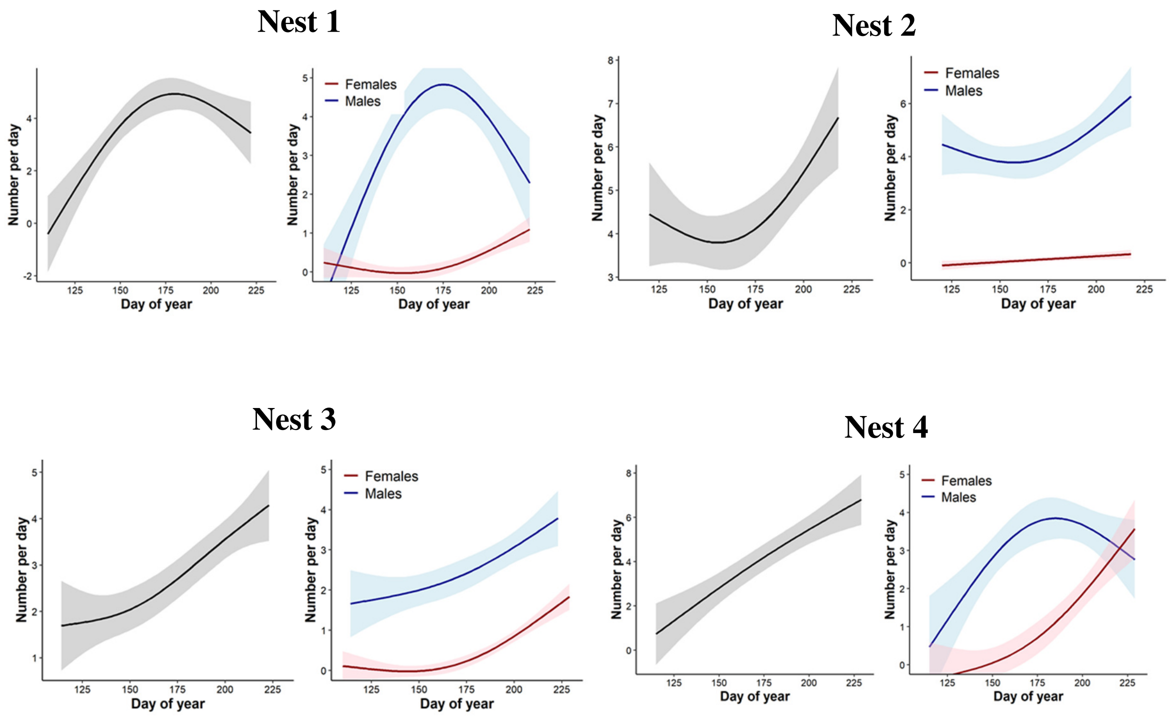

Supplementary Figure 4. General additive models indicating temporal changes in prey deliveries in total and in male and female parents in four studied nests.
