# Supplementary material for: Web-based citizen science as a tool in conservation research: A case study of prey delivery by the Lesser Spotted Eagle
Source: PLoS One. 2022 Jan 26;17(1):e0261655. doi: 10.1371/journal.pone.0261655 (PMC8791511; doi:10.1371/journal.pone.0261655)
Supplement: S2 File — Data used in the analysis; multinomial models indicating dietary differences among studied pairs. (PDF) [file pone.0261655.s002.pdf]

Web-based citizen science as a tool in conservation research: a case study of prey delivery by the Lesser Spotted Eagle

Ülo Väli*, Ana Magalhães

Supporting Information S2 Table

Supplementary Table 1. Data used in the analysis of diet composition and parental roles of the Lesser Spotted Eagle.

Nest	Day	Month	Year	Julian date	Sex	Unidentified Small Mammal	Fish	Frog	Bird	Mole	Vole	Water Vole	Hare	Weasel	NA
1	30	4	2012	120	F	0	0	0	0	0	0	0	0	0	0
1	30	4	2012	120	M	0	0	3	0	0	0	0	0	0	0
1	1	5	2012	121	F	0	0	0	0	0	0	0	0	0	0
1	1	5	2012	121	M	0	0	0	0	0	0	0	0	0	3
1	2	5	2012	122	F	0	0	0	0	0	0	0	0	0	0
1	2	5	2012	122	M	0	0	6	0	0	0	0	0	0	0
1	3	5	2012	123	F	0	0	0	0	0	0	0	0	0	0
1	3	5	2012	123	M	0	0	3	0	0	0	0	0	0	2
1	4	5	2012	124	F	0	0	0	0	0	0	0	0	0	0
1	4	5	2012	124	M	0	0	4	0	0	0	0	0	0	1
1	5	5	2012	125	F	0	0	0	0	0	0	0	0	0	0
1	5	5	2012	125	M	0	0	2	0	0	0	0	0	0	0
1	6	5	2012	126	F	0	0	0	0	0	0	0	0	0	0
1	6	5	2012	126	M	0	0	5	0	0	0	0	0	0	0
1	7	5	2012	127	F	0	0	0	0	0	0	0	0	0	0
1	7	5	2012	127	M	0	0	3	0	0	0	0	0	0	2
1	8	5	2012	128	F	0	0	0	0	0	0	0	0	0	0
1	8	5	2012	128	M	0	0	3	0	0	0	0	0	0	1
1	9	5	2012	129	F	0	0	0	0	0	0	0	0	0	0
1	9	5	2012	129	M	1	0	4	0	0	0	0	0	0	1
1	10	5	2012	130	F	0	0	0	0	0	0	0	0	0	0
1	10	5	2012	130	M	0	0	7	0	0	0	0	0	0	0
1	11	5	2012	131	F	0	0	0	0	0	0	0	0	0	0
1	11	5	2012	131	M	0	0	3	0	0	0	0	0	0	0
1	12	5	2012	132	F	0	0	0	0	0	0	0	0	0	0
1	12	5	2012	132	M	0	0	2	0	0	0	0	0	0	1
1	13	5	2012	133	F	0	0	0	0	0	0	0	0	0	0
1	13	5	2012	133	M	0	0	5	0	0	0	0	0	0	0
1	14	5	2012	134	F	0	0	0	0	0	0	0	0	0	0
1	14	5	2012	134	M	0	0	5	0	0	1	0	0	0	1

1	15	5	2012	135	F	0	0	0	0	0	0	0	0	0	0
1	15	5	2012	135	M	0	0	2	0	0	0	0	0	0	0
1	17	5	2012	137	F	0	0	0	0	0	0	0	0	0	0
1	17	5	2012	137	M	0	0	0	0	0	0	0	0	0	2
1	18	5	2012	138	F	0	0	0	0	0	0	0	0	0	0
1	18	5	2012	138	M	0	0	3	0	0	0	0	0	0	0
1	19	5	2012	139	F	0	0	0	0	0	0	0	0	0	0
1	19	5	2012	139	M	0	0	1	0	0	0	0	0	0	1
1	20	5	2012	140	F	0	0	0	0	0	0	0	0	0	0
1	20	5	2012	140	M	0	0	2	0	0	0	0	0	0	0
1	22	5	2012	142	F	0	0	0	0	0	0	0	0	0	0
1	22	5	2012	142	M	3	0	2	0	0	0	0	0	0	0
1	23	5	2012	143	F	0	0	0	0	0	0	0	0	0	0
1	23	5	2012	143	M	2	0	2	0	0	0	0	0	0	1
1	24	5	2012	144	F	0	0	0	0	0	0	0	0	0	0
1	24	5	2012	144	M	4	0	0	0	0	0	0	0	0	0
1	25	5	2012	145	F	0	0	0	0	0	0	0	0	0	0
1	25	5	2012	145	M	1	0	0	0	0	1	0	0	0	3
1	26	5	2012	146	F	0	0	0	0	0	0	0	0	0	0
1	26	5	2012	146	M	0	0	2	0	0	1	0	0	0	1
1	27	5	2012	147	F	0	0	0	0	0	0	0	0	0	0
1	27	5	2012	147	M	0	0	2	0	0	4	0	0	0	1
1	28	5	2012	148	F	0	0	0	0	0	0	0	0	0	0
1	28	5	2012	148	M	0	0	0	0	0	0	0	0	0	1
1	29	5	2012	149	F	0	0	0	0	0	0	0	0	0	0
1	29	5	2012	149	M	0	0	1	0	0	1	0	0	0	2
1	31	5	2012	151	F	0	0	0	0	0	0	0	0	0	0
1	31	5	2012	151	M	0	0	0	0	0	3	0	0	0	0
1	1	6	2012	152	F	0	0	0	0	0	0	0	0	0	0
1	1	6	2012	152	M	0	0	2	0	0	2	0	0	0	2
1	2	6	2012	153	F	0	0	0	0	0	0	0	0	0	0
1	2	6	2012	153	M	1	0	0	0	0	0	0	0	0	0
1	3	6	2012	154	F	0	0	0	0	0	0	0	0	0	0
1	3	6	2012	154	M	2	0	0	0	0	0	0	0	0	1
1	4	6	2012	155	F	0	0	0	0	0	0	0	0	0	0
1	4	6	2012	155	M	0	0	0	0	0	2	0	0	0	0
1	5	6	2012	156	F	0	0	0	0	0	0	0	0	0	0
1	5	6	2012	156	M	0	0	0	0	0	1	0	0	0	1
1	6	6	2012	157	F	0	0	0	0	0	0	0	0	0	0
1	6	6	2012	157	M	0	0	0	0	0	0	0	0	0	1
1	7	6	2012	158	F	0	0	0	0	0	0	0	0	0	0
1	7	6	2012	158	M	1	0	1	0	0	2	0	0	0	1
1	8	6	2012	159	F	0	0	0	0	0	0	0	0	0	0
1	8	6	2012	159	M	0	0	1	1	0	3	0	0	0	3
1	9	6	2012	160	F	0	0	0	0	0	0	0	0	0	0

1	9	6	2012	160	M	3	0	0	0	0	1	0	0	0	1
1	10	6	2012	161	F	0	0	0	0	0	0	0	0	0	0
1	10	6	2012	161	M	1	0	3	0	0	2	0	0	0	1
1	11	6	2012	162	F	0	0	0	0	0	0	0	0	0	0
1	11	6	2012	162	M	0	0	6	0	0	0	0	0	0	0
1	12	6	2012	163	F	0	0	0	0	0	0	0	0	0	0
1	12	6	2012	163	M	2	0	0	0	0	2	0	0	0	0
1	13	6	2012	164	F	0	0	0	0	0	0	0	0	0	0
1	13	6	2012	164	M	1	0	0	0	1	2	0	0	0	2
1	14	6	2012	165	F	0	0	0	0	0	0	0	0	0	0
1	14	6	2012	165	M	0	0	1	0	0	2	0	0	0	1
1	15	6	2012	166	F	0	0	0	0	0	0	0	0	0	0
1	15	6	2012	166	M	0	0	0	0	0	4	0	0	0	1
1	16	6	2012	167	F	0	0	0	0	0	0	0	0	0	0
1	16	6	2012	167	M	0	0	0	0	0	7	0	0	0	0
1	17	6	2012	168	F	0	0	0	0	0	0	0	0	0	0
1	17	6	2012	168	M	0	0	2	0	0	1	0	0	0	0
1	18	6	2012	169	F	0	0	0	0	0	0	0	0	0	0
1	18	6	2012	169	M	2	0	2	0	0	3	0	0	0	0
1	19	6	2012	170	F	0	0	0	0	0	0	0	0	0	0
1	19	6	2012	170	M	2	0	4	0	0	0	0	0	0	0
1	20	6	2012	171	F	0	0	0	0	0	0	0	0	0	0
1	20	6	2012	171	M	0	0	0	0	0	0	0	0	0	1
1	21	6	2012	172	F	0	0	0	0	0	0	0	0	0	0
1	21	6	2012	172	M	1	0	0	0	0	1	0	0	0	0
1	22	6	2012	173	F	0	0	0	0	0	0	0	0	0	0
1	22	6	2012	173	M	2	0	0	1	0	1	0	0	0	0
1	23	6	2012	174	F	0	0	0	0	0	0	0	0	0	0
1	23	6	2012	174	M	0	0	3	0	0	0	0	0	0	0
1	24	6	2012	175	F	0	0	0	0	0	0	0	0	0	0
1	24	6	2012	175	M	0	0	7	0	0	0	0	0	0	0
1	26	6	2012	177	F	0	0	0	0	0	0	0	0	0	0
1	26	6	2012	177	M	0	0	1	0	0	0	0	0	0	0
1	27	6	2012	178	F	0	0	0	0	0	0	0	0	0	0
1	27	6	2012	178	M	0	0	0	0	0	1	0	0	0	0
1	28	6	2012	179	F	0	0	1	0	0	0	0	0	0	0
1	28	6	2012	179	M	0	0	0	0	1	2	0	0	0	0
1	29	6	2012	180	F	0	0	0	0	0	0	0	0	0	0
1	29	6	2012	180	M	0	0	3	0	0	1	0	0	0	0
1	30	6	2012	181	F	0	0	0	0	0	0	0	0	0	0
1	30	6	2012	181	M	0	0	0	1	0	0	0	0	0	0
1	1	7	2012	182	F	0	0	0	0	0	0	0	0	0	0
1	1	7	2012	182	M	0	0	2	0	0	1	0	0	0	1
1	2	7	2012	183	F	0	0	0	0	0	0	0	0	0	0
1	2	7	2012	183	M	0	0	1	0	0	1	0	0	0	1

1	3	7	2012	184	F	0	0	0	0	0	0	0	0	0	0
1	3	7	2012	184	M	0	0	0	0	0	1	0	0	0	5
1	4	7	2012	185	F	0	0	0	0	0	0	0	0	0	0
1	4	7	2012	185	M	0	0	2	0	0	0	0	0	0	0
1	6	7	2012	187	F	0	0	0	0	0	0	0	0	0	0
1	6	7	2012	187	M	0	0	0	1	0	0	0	0	0	2
1	7	7	2012	188	F	0	0	0	0	0	0	0	0	0	0
1	7	7	2012	188	M	0	0	0	0	1	2	0	0	0	2
1	8	7	2012	189	F	0	0	1	0	0	0	0	0	0	0
1	8	7	2012	189	M	0	0	4	0	0	0	0	0	0	0
1	9	7	2012	190	F	0	0	0	0	0	0	0	0	0	0
1	9	7	2012	190	M	0	0	0	0	2	0	0	0	0	1
1	10	7	2012	191	F	0	0	0	0	0	0	0	0	0	0
1	10	7	2012	191	M	0	0	0	0	0	0	0	0	0	1
1	11	7	2012	192	F	0	0	1	0	0	0	0	0	0	0
1	11	7	2012	192	M	0	0	0	0	0	2	0	0	0	0
1	12	7	2012	193	F	0	0	1	0	0	0	0	0	0	0
1	12	7	2012	193	M	1	0	0	0	1	0	0	0	0	0
1	13	7	2012	194	F	0	0	0	0	0	0	0	0	0	0
1	13	7	2012	194	M	0	0	5	1	0	1	0	0	0	2
1	14	7	2012	195	F	0	0	0	0	0	0	0	0	0	0
1	14	7	2012	195	M	0	1	1	0	1	0	0	0	0	0
1	15	7	2012	196	F	0	0	0	0	0	0	0	0	0	0
1	15	7	2012	196	M	1	0	3	0	0	1	0	0	0	2
1	16	7	2012	197	F	1	0	0	0	0	0	0	0	0	0
1	16	7	2012	197	M	0	0	3	0	1	0	0	0	0	1
1	17	7	2012	198	F	0	0	0	0	0	0	0	0	0	0
1	17	7	2012	198	M	0	0	2	0	0	1	0	0	0	2
1	18	7	2012	199	F	0	0	0	0	0	0	0	0	0	0
1	18	7	2012	199	M	0	0	3	0	1	1	0	0	0	1
1	19	7	2012	200	F	0	0	0	0	0	0	0	0	0	0
1	19	7	2012	200	M	1	0	4	0	0	1	0	0	0	1
1	20	7	2012	201	F	0	0	1	0	0	0	0	0	0	0
1	20	7	2012	201	M	0	0	5	0	0	0	0	0	0	1
1	21	7	2012	202	F	0	0	0	0	0	0	0	0	0	0
1	21	7	2012	202	M	0	0	3	1	0	1	0	0	0	3
1	22	7	2012	203	F	0	0	0	0	0	0	0	0	0	0
1	22	7	2012	203	M	0	0	1	0	0	0	0	0	0	0
1	23	7	2012	204	F	0	0	0	0	0	0	0	0	0	0
1	23	7	2012	204	M	0	0	2	0	0	0	0	0	0	2
1	24	7	2012	205	F	0	0	1	1	0	0	0	0	0	0
1	24	7	2012	205	M	0	0	9	0	0	0	0	0	0	0
1	25	7	2012	206	F	0	0	0	0	0	0	0	0	0	0
1	25	7	2012	206	M	0	0	4	0	0	1	0	0	0	0
1	26	7	2012	207	F	0	0	0	0	0	0	0	0	0	0

1	26	7	2012	207	M	1	0	0	0	0	4	0	0	0	1
1	27	7	2012	208	F	0	0	0	0	0	0	0	0	0	0
1	27	7	2012	208	M	1	0	1	0	2	2	0	0	0	1
1	28	7	2012	209	F	0	0	0	0	0	0	0	0	0	0
1	28	7	2012	209	M	0	0	1	0	1	3	0	0	0	1
1	29	7	2012	210	F	0	0	0	0	0	0	0	0	0	0
1	29	7	2012	210	M	1	0	0	1	1	2	0	0	0	0
1	30	7	2012	211	F	0	0	0	0	0	0	0	0	0	0
1	30	7	2012	211	M	0	0	4	0	0	2	0	0	0	0
1	31	7	2012	212	F	0	0	0	0	0	0	0	0	0	0
1	31	7	2012	212	M	0	0	2	0	0	2	0	0	0	4
1	1	8	2012	213	F	0	0	0	0	0	0	0	0	0	0
1	1	8	2012	213	M	0	0	1	0	0	1	0	0	0	1
1	2	8	2012	214	F	1	0	2	0	0	0	0	0	0	0
1	2	8	2012	214	M	0	0	1	0	0	5	0	0	0	2
1	3	8	2012	215	F	0	0	0	0	0	0	0	0	0	0
1	3	8	2012	215	M	0	0	3	0	0	3	0	0	0	1
1	4	8	2012	216	F	0	0	0	0	0	0	0	0	0	0
1	4	8	2012	216	M	0	0	4	0	1	3	0	0	0	1
1	5	8	2012	217	F	0	0	0	0	0	0	0	0	0	0
1	5	8	2012	217	M	2	0	1	0	2	5	0	0	0	0
1	6	8	2012	218	F	0	0	0	0	0	0	0	0	0	0
1	6	8	2012	218	M	1	0	0	0	0	0	0	0	0	0
2	17	4	2013	107	F	1	0	0	0	0	0	0	0	0	0
2	17	4	2013	107	M	0	0	0	0	0	0	0	0	0	0
2	20	4	2013	110	F	0	0	1	0	0	0	0	0	0	0
2	20	4	2013	110	M	0	0	0	0	0	0	0	0	0	0
2	26	4	2013	116	F	0	0	0	0	0	0	0	0	0	0
2	26	4	2013	116	M	0	0	1	0	0	0	0	0	0	0
2	30	4	2013	120	F	0	0	0	0	0	0	0	0	0	0
2	30	4	2013	120	M	0	0	1	0	0	0	0	0	0	0
2	1	5	2013	121	F	0	0	0	0	0	0	0	0	0	0
2	1	5	2013	121	M	0	0	1	0	0	0	0	0	0	0
2	3	5	2013	123	F	0	0	0	0	0	0	0	0	0	0
2	3	5	2013	123	M	0	0	2	0	0	0	0	0	0	0
2	4	5	2013	124	F	0	0	0	0	0	0	0	0	0	0
2	4	5	2013	124	M	0	0	3	0	0	0	0	0	0	0
2	5	5	2013	125	F	0	0	0	0	0	0	0	0	0	0
2	5	5	2013	125	M	0	0	1	0	0	0	0	0	0	0
2	6	5	2013	126	F	0	0	0	0	0	0	0	0	0	0
2	6	5	2013	126	M	1	0	2	0	0	0	0	0	0	0
2	7	5	2013	127	F	0	0	0	0	0	0	0	0	0	0
2	7	5	2013	127	M	0	0	1	0	0	0	0	0	0	1
2	8	5	2013	128	F	0	0	0	0	0	0	0	0	0	0
2	8	5	2013	128	M	0	0	1	0	0	0	0	0	0	0

2	9	5	2013	129	F	0	0	0	0	0	0	0	0	0	0
2	9	5	2013	129	M	0	0	1	0	0	0	0	0	0	0
2	10	5	2013	130	F	0	0	0	0	0	0	0	0	0	0
2	10	5	2013	130	M	0	0	1	0	0	0	0	0	0	0
2	12	5	2013	132	F	0	0	0	0	0	0	0	0	0	0
2	12	5	2013	132	M	0	0	1	0	0	0	0	0	0	1
2	13	5	2013	133	F	0	0	0	0	0	0	0	0	0	0
2	13	5	2013	133	M	1	0	0	0	0	0	0	0	0	2
2	14	5	2013	134	F	0	0	0	0	0	0	0	0	0	0
2	14	5	2013	134	M	0	0	2	0	0	0	0	0	0	0
2	15	5	2013	135	F	0	0	0	0	0	0	0	0	0	0
2	15	5	2013	135	M	0	0	3	0	0	0	0	0	0	0
2	16	5	2013	136	F	0	0	0	0	0	0	0	0	0	0
2	16	5	2013	136	M	1	0	2	0	0	0	0	0	0	1
2	17	5	2013	137	F	0	0	0	0	0	0	0	0	0	0
2	17	5	2013	137	M	0	0	1	0	0	0	0	0	0	0
2	18	5	2013	138	F	0	0	0	0	0	0	0	0	0	0
2	18	5	2013	138	M	0	0	0	0	0	0	0	0	0	1
2	19	5	2013	139	F	0	0	0	0	0	0	0	0	0	0
2	19	5	2013	139	M	0	0	0	0	0	0	0	0	0	1
2	20	5	2013	140	F	0	0	0	0	0	0	0	0	0	0
2	20	5	2013	140	M	1	0	0	0	0	1	0	0	0	0
2	21	5	2013	141	F	0	0	0	0	0	0	0	0	0	0
2	21	5	2013	141	M	1	0	0	0	0	1	0	0	0	0
2	22	5	2013	142	F	0	0	0	0	0	0	0	0	0	0
2	22	5	2013	142	M	0	0	0	1	0	1	0	0	0	0
2	23	5	2013	143	F	0	0	0	0	0	0	0	0	0	0
2	23	5	2013	143	M	0	0	1	0	0	0	0	0	0	1
2	24	5	2013	144	F	0	0	0	0	0	0	0	0	0	0
2	24	5	2013	144	M	1	0	2	1	0	0	0	0	0	1
2	27	5	2013	147	F	0	0	0	0	0	0	0	0	0	0
2	27	5	2013	147	M	2	0	0	0	0	1	0	0	0	2
2	28	5	2013	148	F	0	0	0	0	0	0	0	0	0	0
2	28	5	2013	148	M	0	0	0	1	0	0	0	0	0	0
2	30	5	2013	150	F	0	0	0	0	0	0	0	0	0	0
2	30	5	2013	150	M	0	0	1	0	0	0	1	0	0	0
2	31	5	2013	151	F	0	0	0	0	0	0	0	0	0	0
2	31	5	2013	151	M	0	0	1	0	0	0	0	0	0	0
2	1	6	2013	152	F	0	0	0	0	0	0	0	0	0	0
2	1	6	2013	152	M	1	0	0	0	0	0	0	0	0	0
2	2	6	2013	153	F	0	0	0	0	0	0	0	0	0	0
2	2	6	2013	153	M	1	0	1	1	0	0	0	0	0	1
2	3	6	2013	154	F	0	0	0	0	0	0	0	0	0	0
2	3	6	2013	154	M	0	0	1	0	0	0	0	0	0	0
2	4	6	2013	155	F	0	0	0	0	0	0	0	0	0	0

2	4	6	2013	155	M	0	0	0	0	0	1	0	0	0	0
2	6	6	2013	157	F	0	0	0	0	0	0	0	0	0	0
2	6	6	2013	157	M	1	0	0	0	0	0	0	0	0	0
2	7	6	2013	158	F	0	0	0	0	0	0	0	0	0	0
2	7	6	2013	158	M	0	0	0	1	0	2	0	0	0	0
2	8	6	2013	159	F	0	0	0	0	0	0	0	0	0	0
2	8	6	2013	159	M	1	0	1	0	0	0	0	0	0	0
2	9	6	2013	160	F	0	0	0	0	0	0	0	0	0	0
2	9	6	2013	160	M	4	0	1	0	0	2	0	0	0	0
2	10	6	2013	161	F	0	0	0	0	0	0	0	0	0	0
2	10	6	2013	161	M	1	0	0	0	0	2	0	0	0	0
2	11	6	2013	162	F	0	0	0	0	0	0	0	0	0	0
2	11	6	2013	162	M	1	0	1	1	0	0	1	0	0	1
2	12	6	2013	163	F	0	0	0	0	0	0	0	0	0	0
2	12	6	2013	163	M	2	0	1	1	1	3	0	0	0	1
2	13	6	2013	164	F	0	0	0	0	0	0	0	0	0	0
2	13	6	2013	164	M	0	0	1	0	0	1	0	0	0	0
2	14	6	2013	165	F	0	0	0	0	0	0	0	0	0	0
2	14	6	2013	165	M	0	0	1	2	0	2	0	1	0	0
2	15	6	2013	166	F	0	0	0	0	0	0	0	0	0	0
2	15	6	2013	166	M	2	0	1	2	0	0	0	0	0	0
2	16	6	2013	167	F	0	0	0	1	0	0	0	0	0	0
2	16	6	2013	167	M	1	0	1	2	2	1	0	0	0	0
2	18	6	2013	169	F	0	0	0	0	0	0	0	0	0	0
2	18	6	2013	169	M	3	0	1	2	1	0	0	0	0	0
2	19	6	2013	170	F	0	0	0	0	0	0	0	0	0	0
2	19	6	2013	170	M	3	0	0	1	2	0	0	0	0	1
2	20	6	2013	171	F	0	0	0	1	0	0	0	0	0	0
2	20	6	2013	171	M	1	0	0	1	0	0	0	0	0	1
2	21	6	2013	172	F	0	0	1	0	0	0	0	0	0	0
2	21	6	2013	172	M	4	0	1	1	0	0	0	0	0	0
2	22	6	2013	173	F	0	0	0	0	0	0	0	0	0	0
2	22	6	2013	173	M	1	0	1	3	0	1	0	0	0	1
2	23	6	2013	174	F	0	0	0	0	0	0	0	0	0	0
2	23	6	2013	174	M	5	0	1	1	0	0	0	0	0	0
2	24	6	2013	175	F	0	0	0	0	0	0	0	0	0	0
2	24	6	2013	175	M	0	0	1	0	2	0	0	0	0	0
2	25	6	2013	176	F	0	0	0	0	0	0	0	0	0	0
2	25	6	2013	176	M	2	0	1	0	0	0	0	0	0	2
2	26	6	2013	177	F	1	0	1	0	0	0	0	0	0	0
2	26	6	2013	177	M	2	0	1	1	1	0	0	0	0	0
2	27	6	2013	178	F	0	0	0	0	0	0	0	0	0	0
2	27	6	2013	178	M	1	0	1	1	3	0	0	0	0	1
2	28	6	2013	179	F	0	0	1	0	0	0	0	0	0	0
2	28	6	2013	179	M	0	0	0	0	1	0	0	0	0	0

2	29	6	2013	180	F	0	0	1	0	0	0	0	0	0	0
2	29	6	2013	180	M	1	0	1	3	1	1	0	0	0	1
2	30	6	2013	181	F	0	0	0	0	0	0	0	0	0	0
2	30	6	2013	181	M	5	0	1	0	1	0	0	0	0	0
2	1	7	2013	182	F	0	0	0	0	0	0	0	0	0	0
2	1	7	2013	182	M	2	0	1	0	0	0	0	0	0	1
2	2	7	2013	183	F	0	0	0	0	0	0	0	0	0	0
2	2	7	2013	183	M	1	0	0	1	2	1	0	0	0	3
2	3	7	2013	184	F	0	0	0	0	0	0	0	0	0	0
2	3	7	2013	184	M	1	0	0	2	0	0	0	0	0	0
2	4	7	2013	185	F	0	0	0	0	0	0	0	0	0	0
2	4	7	2013	185	M	4	0	0	1	0	1	0	0	0	0
2	5	7	2013	186	F	0	0	0	0	0	0	0	0	0	0
2	5	7	2013	186	M	0	0	1	0	0	1	0	0	0	1
2	6	7	2013	187	F	0	0	0	0	0	0	0	0	0	0
2	6	7	2013	187	M	3	0	1	0	0	1	0	0	0	0
2	7	7	2013	188	F	0	0	0	0	0	0	0	0	0	0
2	7	7	2013	188	M	4	0	0	1	0	1	0	0	0	2
2	8	7	2013	189	F	0	0	0	0	0	0	0	0	0	0
2	8	7	2013	189	M	1	0	0	1	0	0	0	0	0	1
2	9	7	2013	190	F	0	0	0	0	0	0	0	0	0	0
2	9	7	2013	190	M	4	0	1	0	1	2	0	0	0	2
2	10	7	2013	191	F	0	0	0	0	0	0	0	0	0	0
2	10	7	2013	191	M	0	0	0	0	0	2	0	0	0	0
2	11	7	2013	192	F	0	0	0	0	0	0	0	0	0	0
2	11	7	2013	192	M	0	0	0	0	0	0	0	0	0	1
2	12	7	2013	193	F	0	0	0	0	0	0	0	0	0	0
2	12	7	2013	193	M	1	0	0	0	0	0	0	0	0	3
2	13	7	2013	194	F	0	0	0	0	0	0	0	0	0	0
2	13	7	2013	194	M	6	0	1	0	0	3	0	0	0	0
2	14	7	2013	195	F	0	0	0	0	0	0	0	0	0	0
2	14	7	2013	195	M	4	0	0	0	1	1	0	0	0	2
2	15	7	2013	196	F	0	0	0	0	0	0	0	0	0	0
2	15	7	2013	196	M	1	0	1	0	1	0	0	0	0	0
2	16	7	2013	197	F	0	0	0	0	0	0	0	0	0	0
2	16	7	2013	197	M	0	0	0	1	0	0	0	0	0	1
2	17	7	2013	198	F	0	0	0	0	0	0	0	0	0	0
2	17	7	2013	198	M	0	0	0	2	0	1	0	0	0	1
2	18	7	2013	199	F	0	0	0	0	0	0	0	0	0	0
2	18	7	2013	199	M	0	0	0	0	0	0	0	0	0	2
2	19	7	2013	200	F	0	0	0	0	0	0	0	0	0	0
2	19	7	2013	200	M	0	0	2	0	0	1	0	0	0	0
2	20	7	2013	201	F	0	0	0	0	0	0	0	0	0	0
2	20	7	2013	201	M	1	0	0	0	0	0	0	0	0	2
2	21	7	2013	202	F	0	0	0	0	0	0	0	0	0	0

2	21	7	2013	202	M	2	0	1	0	0	1	0	0	0	0
2	22	7	2013	203	F	0	0	0	0	0	0	0	0	0	0
2	22	7	2013	203	M	0	0	0	0	2	1	0	0	0	0
2	23	7	2013	204	F	0	0	0	0	0	0	0	0	0	0
2	23	7	2013	204	M	1	0	1	0	0	2	0	0	0	0
2	24	7	2013	205	F	0	0	0	0	0	0	0	0	0	0
2	24	7	2013	205	M	0	0	1	1	0	0	0	0	0	1
2	25	7	2013	206	F	1	0	0	0	0	0	0	0	0	0
2	25	7	2013	206	M	4	0	1	0	1	2	0	0	0	0
2	26	7	2013	207	F	0	0	0	0	0	0	0	0	0	2
2	26	7	2013	207	M	0	0	0	0	0	0	0	0	0	2
2	27	7	2013	208	F	1	0	0	0	0	0	0	0	1	0
2	27	7	2013	208	M	0	0	0	0	0	0	0	0	0	1
2	28	7	2013	209	F	0	0	0	0	0	0	0	0	0	0
2	28	7	2013	209	M	0	0	1	0	0	2	0	0	0	0
2	29	7	2013	210	F	1	0	1	0	0	0	0	0	0	0
2	29	7	2013	210	M	0	0	0	0	0	3	0	0	0	1
2	30	7	2013	211	F	0	0	0	0	0	0	0	0	0	0
2	30	7	2013	211	M	2	0	0	0	0	2	0	0	0	0
2	31	7	2013	212	F	0	0	0	0	0	0	0	0	0	0
2	31	7	2013	212	M	1	0	0	0	0	0	0	0	0	0
2	1	8	2013	213	F	0	0	0	0	0	0	0	0	0	0
2	1	8	2013	213	M	4	0	0	0	1	1	0	0	0	0
2	2	8	2013	214	F	0	0	0	0	1	0	0	0	0	0
2	2	8	2013	214	M	0	0	0	1	1	0	0	0	0	2
2	3	8	2013	215	F	1	0	0	0	0	0	0	0	0	0
2	3	8	2013	215	M	1	0	0	0	0	0	0	0	0	0
2	4	8	2013	216	F	0	0	1	0	1	0	0	0	0	1
2	4	8	2013	216	M	2	0	0	0	0	0	0	0	1	0
2	5	8	2013	217	F	0	0	0	0	0	0	0	0	0	0
2	5	8	2013	217	M	0	0	2	1	0	0	0	0	0	0
2	6	8	2013	218	F	0	0	0	0	0	2	0	0	0	0
2	6	8	2013	218	M	0	0	1	0	0	0	0	0	0	0
2	9	8	2013	221	F	1	0	0	0	0	0	0	0	0	0
2	9	8	2013	221	M	0	0	0	0	0	0	0	0	0	0
2	10	8	2013	222	F	0	0	1	0	1	1	0	0	0	0
2	10	8	2013	222	M	0	0	0	0	0	1	0	0	0	0
3	24	4	2019	114	F	0	0	0	0	0	0	0	0	0	0
3	24	4	2019	114	M	0	0	2	0	0	0	0	0	0	0
3	30	4	2019	120	F	0	0	0	0	0	0	0	0	0	0
3	30	4	2019	120	M	0	0	0	0	0	0	0	0	0	1
3	1	5	2019	121	F	0	0	0	0	0	0	0	0	0	0
3	1	5	2019	121	M	0	0	1	0	0	0	0	0	0	0
3	3	5	2019	123	F	0	0	0	0	0	0	0	0	0	0
3	3	5	2019	123	M	0	0	1	0	0	0	0	0	0	0

3	4	5	2019	124	F	0	0	0	0	0	0	0	0	0	0
3	4	5	2019	124	M	1	0	3	0	0	1	0	0	0	0
3	7	5	2019	127	F	0	0	0	0	0	0	0	0	0	0
3	7	5	2019	127	M	1	0	0	0	2	0	0	0	0	0
3	8	5	2019	128	F	0	0	0	0	0	0	0	0	0	0
3	8	5	2019	128	M	0	0	1	0	0	2	0	0	0	0
3	9	5	2019	129	F	0	0	0	0	0	0	0	0	0	0
3	9	5	2019	129	M	0	0	2	0	0	0	0	0	0	0
3	10	5	2019	130	F	0	0	0	0	0	0	0	0	0	0
3	10	5	2019	130	M	1	0	1	0	0	0	0	0	0	0
3	11	5	2019	131	F	0	0	0	0	0	0	0	0	0	0
3	11	5	2019	131	M	1	0	0	0	0	0	0	0	0	0
3	12	5	2019	132	F	0	0	0	0	0	0	0	0	0	0
3	12	5	2019	132	M	0	0	1	0	0	1	0	0	0	1
3	13	5	2019	133	F	0	0	0	0	0	0	0	0	0	0
3	13	5	2019	133	M	0	0	1	0	0	0	0	0	0	0
3	14	5	2019	134	F	0	0	0	0	0	0	0	0	0	0
3	14	5	2019	134	M	0	0	2	0	0	0	0	0	0	0
3	15	5	2019	135	F	0	0	0	0	0	0	0	0	0	0
3	15	5	2019	135	M	0	0	2	0	0	0	0	0	0	0
3	16	5	2019	136	F	0	0	0	0	0	0	0	0	0	0
3	16	5	2019	136	M	0	0	2	0	0	2	0	0	0	0
3	17	5	2019	137	F	0	0	0	0	0	0	0	0	0	0
3	17	5	2019	137	M	0	0	2	0	0	0	0	0	0	0
3	18	5	2019	138	F	0	0	0	0	0	0	0	0	0	0
3	18	5	2019	138	M	0	0	1	0	0	0	1	0	0	0
3	19	5	2019	139	F	0	0	0	0	0	0	0	0	0	0
3	19	5	2019	139	M	0	0	0	0	0	2	0	0	0	0
3	20	5	2019	140	F	0	0	0	0	0	0	0	0	0	0
3	20	5	2019	140	M	2	0	0	0	0	0	0	0	0	0
3	21	5	2019	141	F	0	0	0	0	0	0	0	0	0	0
3	21	5	2019	141	M	0	0	0	0	0	1	0	0	0	0
3	22	5	2019	142	F	0	0	0	0	0	0	0	0	0	0
3	22	5	2019	142	M	0	0	0	0	0	2	0	0	0	0
3	23	5	2019	143	F	0	0	0	0	0	0	0	0	0	0
3	23	5	2019	143	M	0	0	0	0	0	1	0	0	0	0
3	25	5	2019	145	F	0	0	0	0	0	0	0	0	0	0
3	25	5	2019	145	M	0	0	0	0	0	1	0	0	0	0
3	26	5	2019	146	F	0	0	0	0	0	0	0	0	0	0
3	26	5	2019	146	M	0	0	0	1	0	0	0	0	0	0
3	28	5	2019	148	F	0	0	0	0	0	0	0	0	0	0
3	28	5	2019	148	M	0	0	0	0	0	0	2	0	0	0
3	31	5	2019	151	F	0	0	0	0	0	0	0	0	0	0
3	31	5	2019	151	M	0	0	0	0	0	0	1	0	0	0
3	2	6	2019	153	F	0	0	0	0	0	0	0	0	0	0

3	2	6	2019	153	M	0	0	0	0	0	2	0	0	0	0
3	3	6	2019	154	F	0	0	0	0	0	0	0	0	0	0
3	3	6	2019	154	M	1	0	0	0	0	0	0	0	0	0
3	6	6	2019	157	F	0	0	0	0	0	0	0	0	0	0
3	6	6	2019	157	M	1	0	0	0	0	0	0	0	0	0
3	8	6	2019	159	F	0	0	0	0	0	0	0	0	0	0
3	8	6	2019	159	M	1	0	0	0	1	0	0	0	0	0
3	9	6	2019	160	F	0	0	0	0	0	0	0	0	0	0
3	9	6	2019	160	M	0	0	0	0	1	0	0	0	0	0
3	10	6	2019	161	F	0	0	0	0	0	0	0	0	0	0
3	10	6	2019	161	M	1	0	0	0	0	0	1	0	0	0
3	11	6	2019	162	F	0	0	0	0	0	0	0	0	0	0
3	11	6	2019	162	M	2	0	0	0	0	0	0	0	0	1
3	12	6	2019	163	F	0	0	0	0	0	0	0	0	0	0
3	12	6	2019	163	M	1	0	0	0	0	0	0	0	0	0
3	13	6	2019	164	F	0	0	0	0	0	0	0	0	0	0
3	13	6	2019	164	M	0	0	0	0	0	0	0	0	0	1
3	14	6	2019	165	F	0	0	0	0	0	0	0	0	0	0
3	14	6	2019	165	M	3	0	0	0	1	0	0	0	0	0
3	15	6	2019	166	F	0	0	0	0	0	0	0	0	0	0
3	15	6	2019	166	M	3	0	0	0	0	0	0	0	0	0
3	16	6	2019	167	F	0	0	0	0	0	0	0	0	0	0
3	16	6	2019	167	M	3	0	0	0	0	0	0	0	0	0
3	17	6	2019	168	F	0	0	1	0	0	0	0	0	0	0
3	17	6	2019	168	M	3	0	1	0	0	0	0	0	0	0
3	18	6	2019	169	F	0	0	0	0	0	0	0	0	0	0
3	18	6	2019	169	M	0	0	1	0	0	0	0	0	0	1
3	19	6	2019	170	F	0	0	0	0	0	0	0	0	0	0
3	19	6	2019	170	M	1	0	0	0	0	0	0	0	0	0
3	20	6	2019	171	F	0	0	1	0	0	0	0	0	0	0
3	20	6	2019	171	M	3	0	0	0	0	0	0	0	0	0
3	21	6	2019	172	F	1	0	0	0	0	0	0	0	0	0
3	21	6	2019	172	M	2	0	1	0	0	0	0	0	0	0
3	23	6	2019	174	F	0	0	0	0	0	0	0	0	0	0
3	23	6	2019	174	M	0	0	0	0	0	2	0	0	0	0
3	24	6	2019	175	F	0	0	0	0	0	0	0	0	0	0
3	24	6	2019	175	M	1	0	0	0	0	0	0	0	0	0
3	25	6	2019	176	F	0	0	0	0	0	0	0	0	0	0
3	25	6	2019	176	M	3	0	0	0	0	0	0	0	0	0
3	26	6	2019	177	F	0	0	0	0	0	0	0	0	0	0
3	26	6	2019	177	M	0	0	1	1	0	0	0	0	0	1
3	27	6	2019	178	F	0	0	1	0	0	0	0	0	0	0
3	27	6	2019	178	M	0	0	0	0	0	0	0	0	0	0
3	28	6	2019	179	F	0	0	0	0	0	0	0	0	0	0
3	28	6	2019	179	M	3	0	0	0	0	0	0	0	0	0

3	29	6	2019	180	F	0	0	0	0	0	0	0	0	0	0
3	29	6	2019	180	M	1	0	0	0	0	0	0	0	0	0
3	30	6	2019	181	F	0	0	0	0	0	0	0	0	0	0
3	30	6	2019	181	M	0	0	0	0	0	0	0	0	0	1
3	2	7	2019	183	F	2	0	1	1	0	0	0	0	0	0
3	2	7	2019	183	M	2	0	1	0	0	0	0	0	0	0
3	3	7	2019	184	F	0	0	0	0	0	0	0	0	0	0
3	3	7	2019	184	M	2	0	0	1	0	0	0	0	0	0
3	4	7	2019	185	F	0	0	0	0	0	0	0	0	0	0
3	4	7	2019	185	M	2	0	0	0	0	0	0	0	0	2
3	5	7	2019	186	F	0	0	0	0	0	0	0	0	0	0
3	5	7	2019	186	M	2	0	0	1	0	0	0	0	0	0
3	6	7	2019	187	F	1	0	0	0	0	0	0	0	0	0
3	6	7	2019	187	M	1	0	0	0	0	0	0	0	0	0
3	7	7	2019	188	F	0	0	0	0	0	0	0	0	0	1
3	7	7	2019	188	M	0	0	0	1	0	0	0	0	0	0
3	8	7	2019	189	F	0	0	0	0	0	0	0	0	0	0
3	8	7	2019	189	M	0	0	0	0	0	0	0	0	0	1
3	9	7	2019	190	F	1	0	0	0	0	0	0	0	0	0
3	9	7	2019	190	M	1	0	0	0	0	0	0	0	0	0
3	10	7	2019	191	F	0	0	0	0	0	0	0	0	0	0
3	10	7	2019	191	M	0	0	0	1	0	0	0	0	0	2
3	11	7	2019	192	F	1	0	0	0	0	0	0	0	0	0
3	11	7	2019	192	M	2	0	0	1	0	0	0	0	0	2
3	12	7	2019	193	F	0	0	0	0	0	0	0	0	0	0
3	12	7	2019	193	M	1	0	0	0	0	0	1	0	0	1
3	13	7	2019	194	F	0	0	0	0	0	0	0	0	0	0
3	13	7	2019	194	M	2	0	0	0	0	0	0	0	0	2
3	14	7	2019	195	F	0	0	0	0	0	0	0	0	0	0
3	14	7	2019	195	M	1	0	0	0	0	0	0	0	0	2
3	15	7	2019	196	F	0	0	0	0	0	0	0	0	0	0
3	15	7	2019	196	M	3	0	0	0	0	0	0	0	0	1
3	16	7	2019	197	F	0	0	0	0	0	0	0	0	0	0
3	16	7	2019	197	M	2	0	0	0	0	0	0	0	0	0
3	17	7	2019	198	F	0	0	0	0	0	0	0	0	0	0
3	17	7	2019	198	M	2	0	0	1	0	0	0	0	0	4
3	18	7	2019	199	F	0	0	0	0	0	0	0	0	0	0
3	18	7	2019	199	M	5	0	0	0	0	0	0	0	0	0
3	19	7	2019	200	F	0	0	0	0	0	0	0	0	0	0
3	19	7	2019	200	M	2	0	0	0	1	0	0	0	0	1
3	20	7	2019	201	F	1	0	0	0	0	0	0	0	0	1
3	20	7	2019	201	M	3	0	0	0	0	0	0	0	0	0
3	21	7	2019	202	F	1	0	0	0	0	0	0	0	0	0
3	21	7	2019	202	M	5	0	0	0	0	0	0	0	0	0
3	22	7	2019	203	F	1	0	0	0	0	0	0	0	0	0

3	22	7	2019	203	M	2	0	1	1	0	0	0	0	0	0
3	23	7	2019	204	F	0	0	0	0	0	0	0	0	0	0
3	23	7	2019	204	M	2	0	0	0	0	0	0	0	0	0
3	24	7	2019	205	F	0	0	0	0	0	0	0	0	0	0
3	24	7	2019	205	M	5	0	2	0	0	0	0	0	0	1
3	25	7	2019	206	F	0	0	0	0	0	0	0	0	0	0
3	25	7	2019	206	M	0	0	0	0	0	0	0	0	0	1
3	26	7	2019	207	F	0	0	0	0	0	0	0	0	0	0
3	26	7	2019	207	M	3	0	0	0	0	0	0	0	0	0
3	27	7	2019	208	F	1	0	0	0	0	0	0	0	0	0
3	27	7	2019	208	M	3	0	0	0	0	0	0	0	0	0
3	28	7	2019	209	F	0	0	1	0	0	0	0	0	0	0
3	28	7	2019	209	M	3	0	1	0	0	0	0	0	0	1
3	29	7	2019	210	F	0	0	1	0	0	0	0	0	0	0
3	29	7	2019	210	M	5	0	1	0	0	0	0	0	0	0
3	30	7	2019	211	F	0	0	0	1	0	0	0	0	0	0
3	30	7	2019	211	M	2	0	0	0	0	0	0	0	0	0
3	31	7	2019	212	F	1	0	0	0	0	0	0	0	0	0
3	31	7	2019	212	M	3	0	0	0	0	0	0	0	0	0
3	1	8	2019	213	F	0	0	0	0	0	0	0	0	0	0
3	1	8	2019	213	M	0	0	0	1	0	0	0	0	0	0
3	2	8	2019	214	F	0	0	0	0	0	0	0	0	0	1
3	2	8	2019	214	M	1	0	0	0	0	0	0	0	0	0
3	3	8	2019	215	F	0	0	0	0	0	0	0	0	0	0
3	3	8	2019	215	M	1	0	0	0	0	0	0	0	0	0
3	4	8	2019	216	F	0	0	0	0	0	0	0	0	0	0
3	4	8	2019	216	M	2	0	0	0	0	0	0	0	0	2
3	5	8	2019	217	F	0	0	0	0	0	0	0	0	0	0
3	5	8	2019	217	M	4	0	1	0	0	0	0	0	0	0
3	6	8	2019	218	F	1	0	0	0	0	0	0	0	0	0
3	6	8	2019	218	M	1	0	0	0	0	0	0	0	0	0
3	7	8	2019	219	F	0	0	1	0	0	0	0	0	0	0
3	7	8	2019	219	M	4	0	0	0	0	0	0	0	0	0
3	8	8	2019	220	F	0	0	0	0	0	0	0	0	0	0
3	8	8	2019	220	M	3	0	2	0	0	0	0	0	0	0
3	9	8	2019	221	F	0	0	0	0	0	0	0	0	0	0
3	9	8	2019	221	M	4	0	0	0	0	1	0	0	0	0
3	10	8	2019	222	F	0	0	0	0	0	0	0	0	0	0
3	10	8	2019	222	M	5	0	0	0	0	0	0	0	0	0
3	11	8	2019	223	F	0	0	1	0	0	0	0	0	0	0
3	11	8	2019	223	M	2	0	0	0	0	0	0	0	0	0
4	24	4	2020	115	F	0	0	0	0	0	0	0	0	0	0
4	24	4	2020	115	M	1	0	0	0	0	0	0	0	0	0
4	5	5	2020	126	F	0	0	0	0	0	0	0	0	0	0
4	5	5	2020	126	M	1	0	1	0	0	0	0	0	0	2

4	6	5	2020	127	F	0	0	0	0	0	0	0	0	0	0
4	6	5	2020	127	M	1	0	0	0	0	0	0	0	0	0
4	7	5	2020	128	F	0	0	0	0	0	0	0	0	0	0
4	7	5	2020	128	M	0	0	1	0	0	0	0	0	0	0
4	8	5	2020	129	F	0	0	0	0	0	0	0	0	0	0
4	8	5	2020	129	M	0	0	2	0	0	0	0	0	0	0
4	9	5	2020	130	F	0	0	0	0	0	0	0	0	0	0
4	9	5	2020	130	M	0	0	0	0	1	0	0	0	0	0
4	10	5	2020	131	F	0	0	0	0	0	0	0	0	0	0
4	10	5	2020	131	M	0	0	0	0	0	0	0	0	0	1
4	11	5	2020	132	F	0	0	0	0	0	0	0	0	0	0
4	11	5	2020	132	M	0	0	2	0	0	0	0	0	0	0
4	12	5	2020	133	F	0	0	0	0	0	0	0	0	0	0
4	12	5	2020	133	M	0	0	1	0	0	3	0	0	0	2
4	14	5	2020	135	F	0	0	0	0	0	0	0	0	0	0
4	14	5	2020	135	M	0	0	2	0	0	0	0	0	0	1
4	15	5	2020	136	F	0	0	0	0	0	0	0	0	0	0
4	15	5	2020	136	M	0	0	0	0	0	1	0	0	0	0
4	16	5	2020	137	F	0	0	0	0	0	0	0	0	0	0
4	16	5	2020	137	M	0	0	0	0	0	1	0	0	0	0
4	18	5	2020	139	F	0	0	0	0	0	0	0	0	0	0
4	18	5	2020	139	M	0	0	0	0	0	1	0	0	0	0
4	19	5	2020	140	F	0	0	0	0	0	0	0	0	0	0
4	19	5	2020	140	M	0	0	0	0	0	1	0	0	0	0
4	20	5	2020	141	F	0	0	0	0	0	0	0	0	0	0
4	20	5	2020	141	M	1	0	0	0	0	0	0	0	0	0
4	22	5	2020	143	F	0	0	0	0	0	0	0	0	0	0
4	22	5	2020	143	M	1	0	1	0	0	1	0	0	0	0
4	23	5	2020	144	F	0	0	0	0	0	0	0	0	0	0
4	23	5	2020	144	M	1	0	1	0	0	0	0	0	0	0
4	24	5	2020	145	F	0	0	0	0	0	0	0	0	0	0
4	24	5	2020	145	M	1	0	0	0	0	1	0	0	0	0
4	25	5	2020	146	F	0	0	0	0	0	0	0	0	0	0
4	25	5	2020	146	M	1	0	0	0	0	0	0	0	0	0
4	26	5	2020	147	F	0	0	0	0	0	0	0	0	0	0
4	26	5	2020	147	M	1	0	1	0	0	0	0	0	0	0
4	27	5	2020	148	F	0	0	0	0	0	0	0	0	0	0
4	27	5	2020	148	M	0	0	1	0	0	1	0	0	0	0
4	28	5	2020	149	F	0	0	0	0	0	0	0	0	0	0
4	28	5	2020	149	M	1	0	1	0	0	0	0	0	0	0
4	29	5	2020	150	F	0	0	0	0	0	0	0	0	0	0
4	29	5	2020	150	M	0	0	1	0	0	2	0	0	0	0
4	1	6	2020	153	F	0	0	0	0	0	0	0	0	0	0
4	1	6	2020	153	M	0	0	0	0	0	1	0	0	0	0
4	2	6	2020	154	F	0	0	0	0	0	0	0	0	0	0

4	2	6	2020	154	M	0	0	0	0	0	1	0	0	0	0
4	3	6	2020	155	F	0	0	0	0	0	0	0	0	0	0
4	3	6	2020	155	M	0	0	0	0	0	1	0	0	0	0
4	5	6	2020	157	F	0	0	0	0	0	0	0	0	0	0
4	5	6	2020	157	M	0	0	0	0	0	1	0	0	0	0
4	6	6	2020	158	F	0	0	0	0	0	0	0	0	0	0
4	6	6	2020	158	M	1	0	0	0	0	1	0	0	0	1
4	7	6	2020	159	F	0	0	0	0	0	0	0	0	0	0
4	7	6	2020	159	M	0	0	0	0	0	1	0	0	0	0
4	8	6	2020	160	F	0	0	0	0	0	0	0	0	0	0
4	8	6	2020	160	M	0	0	0	0	0	2	0	0	0	0
4	9	6	2020	161	F	0	0	0	0	0	0	0	0	0	0
4	9	6	2020	161	M	0	0	0	0	0	2	0	0	0	0
4	10	6	2020	162	F	0	0	0	0	0	0	0	0	0	0
4	10	6	2020	162	M	2	0	0	0	0	1	0	0	0	0
4	11	6	2020	163	F	0	0	0	0	0	0	0	0	0	0
4	11	6	2020	163	M	1	0	0	0	0	4	0	0	0	0
4	12	6	2020	164	F	0	0	0	0	0	0	0	0	0	0
4	12	6	2020	164	M	0	0	1	0	0	4	0	0	0	0
4	13	6	2020	165	F	0	0	0	0	0	0	0	0	0	0
4	13	6	2020	165	M	0	0	0	0	0	4	0	0	0	0
4	14	6	2020	166	F	0	0	0	0	0	0	0	0	0	0
4	14	6	2020	166	M	0	0	0	0	0	4	0	0	0	0
4	15	6	2020	167	F	0	0	0	0	0	0	0	0	0	0
4	15	6	2020	167	M	3	0	0	1	0	0	0	0	0	0
4	16	6	2020	168	F	0	0	0	0	0	0	0	0	0	0
4	16	6	2020	168	M	2	0	2	0	0	2	0	0	0	0
4	17	6	2020	169	F	0	0	0	0	0	0	0	0	0	0
4	17	6	2020	169	M	1	0	0	0	0	4	0	0	0	0
4	18	6	2020	170	F	0	0	0	0	0	0	0	0	0	0
4	18	6	2020	170	M	0	0	0	0	0	4	0	0	0	0
4	19	6	2020	171	F	0	0	0	0	0	0	0	0	0	0
4	19	6	2020	171	M	1	0	1	3	0	1	0	0	0	0
4	20	6	2020	172	F	0	0	0	0	0	0	0	0	0	0
4	20	6	2020	172	M	0	0	2	2	0	0	0	0	0	0
4	21	6	2020	173	F	0	0	0	0	0	0	0	0	0	0
4	21	6	2020	173	M	1	0	0	0	0	1	0	0	0	0
4	22	6	2020	174	F	0	0	0	0	0	0	0	0	0	0
4	22	6	2020	174	M	0	0	3	0	0	2	0	0	0	0
4	23	6	2020	175	F	1	0	0	0	0	0	0	0	0	0
4	23	6	2020	175	M	1	0	1	2	0	8	0	0	0	0
4	24	6	2020	176	F	0	0	0	0	0	0	0	0	0	0
4	24	6	2020	176	M	1	0	0	1	0	3	0	0	0	0
4	25	6	2020	177	F	0	0	0	0	0	0	0	0	0	0
4	25	6	2020	177	M	0	0	0	0	0	3	0	0	0	0

4	26	6	2020	178	F	0	0	0	0	0	1	0	0	0	0
4	26	6	2020	178	M	0	0	0	1	0	1	0	0	0	1
4	27	6	2020	179	F	0	0	1	0	0	0	0	0	0	0
4	27	6	2020	179	M	0	0	1	0	0	2	0	0	0	0
4	28	6	2020	180	F	0	0	0	1	0	2	0	0	0	0
4	28	6	2020	180	M	0	0	0	0	0	4	0	0	0	0
4	29	6	2020	181	F	0	0	0	0	0	0	0	0	0	0
4	29	6	2020	181	M	0	0	0	0	0	3	0	0	0	0
4	30	6	2020	182	F	0	0	0	0	0	0	0	0	0	0
4	30	6	2020	182	M	0	0	1	2	0	1	0	0	0	1
4	1	7	2020	183	F	0	0	0	1	0	0	0	0	0	0
4	1	7	2020	183	M	0	0	3	1	0	0	0	0	0	0
4	2	7	2020	184	F	0	0	1	0	0	0	0	0	0	0
4	2	7	2020	184	M	0	0	0	1	0	3	0	0	0	0
4	3	7	2020	185	F	0	0	0	0	0	0	0	0	0	0
4	3	7	2020	185	M	0	0	2	0	0	1	0	0	0	0
4	4	7	2020	186	F	0	0	2	0	0	0	0	0	0	0
4	4	7	2020	186	M	0	0	3	0	0	0	0	0	0	0
4	5	7	2020	187	F	0	0	3	0	0	0	0	0	0	0
4	5	7	2020	187	M	0	0	2	0	1	0	0	0	0	1
4	6	7	2020	188	F	0	0	1	0	0	0	0	0	0	0
4	6	7	2020	188	M	0	0	4	0	1	0	0	0	0	0
4	7	7	2020	189	F	0	0	1	0	0	0	0	0	0	0
4	7	7	2020	189	M	0	0	1	1	0	3	0	0	0	0
4	8	7	2020	190	F	0	0	0	1	0	0	0	0	0	0
4	8	7	2020	190	M	0	0	0	0	0	1	0	0	0	0
4	12	7	2020	194	F	0	0	0	0	0	0	0	0	0	0
4	12	7	2020	194	M	0	0	0	0	0	7	0	0	0	0
4	13	7	2020	195	F	0	0	1	0	0	3	1	0	0	0
4	13	7	2020	195	M	1	0	0	0	0	7	0	0	0	1
4	14	7	2020	196	F	0	0	0	0	0	0	0	0	0	0
4	14	7	2020	196	M	0	0	0	0	0	1	0	0	0	0
4	15	7	2020	197	F	0	0	0	0	1	0	0	0	0	0
4	15	7	2020	197	M	0	0	0	1	0	5	0	0	0	0
4	16	7	2020	198	F	0	0	0	0	0	0	0	0	0	0
4	16	7	2020	198	M	1	0	0	0	0	0	0	0	0	0
4	17	7	2020	199	F	0	0	0	0	0	2	0	0	0	0
4	17	7	2020	199	M	0	0	1	0	1	3	0	0	0	0
4	18	7	2020	200	F	0	0	0	0	0	2	0	0	0	0
4	18	7	2020	200	M	0	0	0	1	0	3	0	0	0	0
4	19	7	2020	201	F	0	0	0	0	0	0	0	0	0	0
4	19	7	2020	201	M	0	0	0	0	0	2	0	0	0	0
4	20	7	2020	202	F	0	0	2	0	0	0	0	0	0	0
4	20	7	2020	202	M	0	0	1	0	0	0	1	0	0	2
4	21	7	2020	203	F	0	0	1	0	0	1	0	0	0	0

4	21	7	2020	203	M	0	0	2	0	0	1	0	0	0	0
4	22	7	2020	204	F	0	0	2	0	0	1	0	0	0	0
4	22	7	2020	204	M	1	0	1	0	1	2	0	0	0	1
4	23	7	2020	205	F	0	0	0	0	0	0	0	0	0	0
4	23	7	2020	205	M	0	0	2	0	0	2	0	0	0	0
4	24	7	2020	206	F	0	0	3	0	0	0	0	0	0	0
4	24	7	2020	206	M	0	0	0	0	0	1	0	0	0	1
4	25	7	2020	207	F	0	0	3	0	0	0	0	0	0	0
4	25	7	2020	207	M	0	0	1	0	0	0	0	0	0	0
4	26	7	2020	208	F	0	0	0	0	0	5	0	0	0	0
4	26	7	2020	208	M	1	0	0	0	0	1	0	0	0	2
4	27	7	2020	209	F	0	0	0	0	0	4	0	0	0	0
4	27	7	2020	209	M	0	0	0	0	0	2	0	0	0	0
4	28	7	2020	210	F	0	0	6	0	0	3	0	0	0	0
4	28	7	2020	210	M	0	0	0	1	0	2	0	0	0	0
4	29	7	2020	211	F	0	0	2	0	0	0	0	0	0	0
4	29	7	2020	211	M	0	0	0	0	0	0	0	0	0	1
4	30	7	2020	212	F	0	0	3	0	0	0	0	0	0	0
4	30	7	2020	212	M	0	0	0	0	0	0	0	0	0	0
4	31	7	2020	213	F	0	0	0	0	0	1	0	0	0	0
4	31	7	2020	213	M	0	0	0	0	0	0	0	0	0	0
4	1	8	2020	214	F	0	0	2	0	0	5	0	0	0	0
4	1	8	2020	214	M	1	0	1	0	0	1	0	0	0	0
4	2	8	2020	215	F	0	0	0	0	0	7	0	0	0	0
4	2	8	2020	215	M	0	0	0	0	0	6	0	0	0	0
4	3	8	2020	216	F	0	0	0	0	0	2	0	0	0	0
4	3	8	2020	216	M	1	0	1	0	0	1	0	0	0	0
4	4	8	2020	217	F	0	0	0	0	0	2	0	0	0	1
4	4	8	2020	217	M	1	0	0	0	0	3	0	0	0	2
4	5	8	2020	218	F	0	0	0	0	0	4	0	0	0	0
4	5	8	2020	218	M	0	0	0	0	0	1	0	0	0	0
4	6	8	2020	219	F	0	0	0	0	0	1	0	0	0	0
4	6	8	2020	219	M	0	0	0	0	0	3	0	0	0	0
4	7	8	2020	220	F	0	0	0	0	0	5	0	0	0	0
4	7	8	2020	220	M	0	0	0	0	0	4	0	0	0	0
4	8	8	2020	221	F	0	0	0	0	0	4	0	0	0	0
4	8	8	2020	221	M	0	0	0	1	0	2	0	0	0	0
4	9	8	2020	222	F	0	0	0	0	0	0	0	0	0	0
4	9	8	2020	222	M	0	0	0	0	0	0	1	0	0	0
4	10	8	2020	223	F	0	0	0	0	0	2	0	0	0	0
4	10	8	2020	223	M	0	0	0	0	0	2	0	0	0	0
4	11	8	2020	224	F	0	0	1	0	0	1	0	0	0	0
4	11	8	2020	224	M	0	0	1	1	0	1	0	1	0	0
4	12	8	2020	225	F	0	0	0	0	0	0	0	0	0	0
4	12	8	2020	225	M	0	0	1	0	0	5	0	0	0	0

4	14	8	2020	227	F	0	0	0	0	0	0	0	0	0	0
4	14	8	2020	227	M	0	0	0	0	0	2	0	0	0	0
4	16	8	2020	229	F	0	0	0	0	0	4	0	0	0	0
4	16	8	2020	229	M	0	0	1	0	0	4	0	0	0	0

Supplementary Table 2. Estimates (±SE) of multinomial models indicating significant differences among three studied pairs (comparisons with nests 1, 2 and 4, respectively). Significant differences are indicated in bold, **P* < 0.05; ***P* < 0.01; ****P* < 0.001.

**Compared with nest 1**	Nest 2	Nest 3	Nest 4
(Intercept)	0.15 ± 0.14	0.23 ± 0.13	-0.05 ± 0.14
Frogs	**-0.51 ± 0.11*****	**-0.77 ± 0.14 *****	**-0.36 ± 0.10 *****
Birds	**1.31 ± 0.38*****	0.42 ± 0.45	**0.80 ± 0.40***
Voles	0.01 ± 0.08	0.14 ± 0.08	**0.29 ± 0.08 *****
Moles	0.26 ± 0.30	-0.83 ± 0.46	**-1.17 ± 0.47 ***
Other	1.36 ± 1.09	1.71 ± 1.08	1.29 ± 1.12
**Compared with nest 2**	Nest 1	Nest 3	Nest 4
(Intercept)	-0.15 ± 0.14	0.08 ± 0.13	-0.21 ± 0.14
Frogs	**0.51 ± 0.11*****	-0.25 ± 0.16	0.16 ± 0.13
Birds	**-1.31 ± 0.38*****	**-0.89 ± 0.32****	**-0.51 ± 0.25***
Voles	-0.01 ± 0.08	0.13 ± 0.08	**0.29 ± 0.07 *****
Moles	-0.26 ± 0.30	**-1.09 ± 0.43***	**-1.43 ± 0.45****
Other	-1.36 ± 1.09	0.36 ± 0.60	-0.07 ± 0.66
**Compared with nest 4**	Nest 1	Nest 2	Nest 3
(Intercept)	0.06 ± 0.14	0.21 ± 0.14	**0.30 ± 0.13 ***
Frogs	**0.36 ± 0.10 *****	-0.16 ± 0.13	**-0.41 ± 0.15 ****
Birds	**-0.80 ± 0.40 ***	**0.51 ± 0.25 ***	-0.38 ± 0.34
Voles	**-0.30 ± 0.08 *****	**-0.29 ± 0.07 *****	**-0.15 ± 0.07 ***
Moles	1.18 ± 0.47	**1.44 ± 0.45****	-0.35 ± 0.55
Other	-1.29 ± 1.11	0.07 ± 0.66	0.43 ± 0.63
